# Growth cone repulsion to Netrin-1 depends on lipid raft microdomains enriched in UNC5 receptors

**DOI:** 10.1007/s00018-020-03663-z

**Published:** 2020-10-23

**Authors:** Marc Hernaiz-Llorens, Cristina Roselló-Busquets, Nela Durisic, Adam Filip, Fausto Ulloa, Ramón Martínez-Mármol, Eduardo Soriano

**Affiliations:** 1 Department of Cell Biology, Physiology and Immunology, Faculty of Biology and Institute of Neurosciences, University of Barcelona, 08028 Barcelona, Spain; 2 Centro de Investigación Biomédica en Red Sobre Enfermedades Neurodegenerativas (CIBERNED), ISCIII, 28031 Madrid, Spain; 3 Vall D´Hebron Institute of Research (VHIR), 08035 Barcelona, Spain; 4 Institució Catalana de Recerca I Estudis Avançats (ICREA), 08010 Barcelona, Spain; 5 Queensland Brain Institute (QBI), The University of Queensland, St Lucia Campus, Brisbane, QLD 4072 Australia; 6 Clem Jones Centre for Ageing Dementia Research (CJCADR), Queensland Brain Institute (QBI), The University of Queensland, St Lucia Campus, Brisbane, QLD 4072 Australia; 7 Nencki Institute of Experimental Biology, Polish Academy of Sciences, 3 Pasteur Street, 02-093 Warsaw, Poland

**Keywords:** UNC5, Netrin-1, Cerebellar EGL neurons, Axonal repulsion, Lipid raft microdomain, Single particle tracking

## Abstract

During brain development, Uncoordinated locomotion 5 (UNC5) receptors control axonal extension through their sensing of the guidance molecule Netrin-1. The correct positioning of receptors into cholesterol-enriched membrane raft microdomains is crucial for the efficient transduction of the recognized signals. However, whether such microdomains are required for the appropriate axonal guidance mediated by UNC5 receptors remains unknown. Here, we combine the use of confocal microscopy, live-cell FRAP analysis and single-particle tracking PALM to characterize the distribution of UNC5 receptors into raft microdomains, revealing differences in their membrane mobility properties. Using pharmacological and genetic approaches in primary neuronal cultures and brain cerebellar explants we further demonstrate that disrupting raft microdomains inhibits the chemorepulsive response of growth cones and axons against Netrin-1. Together, our findings indicate that the distribution of all UNC5 receptors into cholesterol-enriched raft microdomains is heterogeneous and that the specific localization has functional consequences for the axonal chemorepulsion against Netrin-1.

**Electronic supplementary material:**

The online version of this article (10.1007/s00018-020-03663-z) contains supplementary material, which is available to authorized users.

## Introduction

The establishment of proper neuronal connections relies on the ability of axons to locate and reach their target during neural development. Axons must elongate to find and contact their partners, which can be very distant from the soma of the neuron. Thus, axons need specific extracellular cues to properly navigate towards their final destination. The leading edge of the axon is known as the growth cone, which is the major motile structure that senses extracellular signals. Netrins are guidance cues involved in a variety of cellular processes such as cell survival [1], cell migration [2] and neuronal axon guidance [3]. In vertebrates, four netrins (Netrin1-4) have been identified [4], and are recognized by four different families of receptors that mediate the guidance functions of Netrins: Deleted in colorectal cancer (DCC) [5], neogenin/DCC like molecule [6], Down syndrome cell adhesion molecule (DSCAM) [7] and Uncoordinated locomotion 5 (UNC5 A to D) [8]. UNC5(A–D) are single-spanning transmembrane proteins whose extracellular region consists of two immunoglobulin domains (Ig) that bind Netrin-1 and two type I thrombospondin domains (TSP) [9]. A ZU-5 domain, a DCC-binding (DB) motif and a death domain (DD) motif comprise the cytoplasmic region [10].

Netrin-1 acts as a chemotactic diffusible molecule, as well as an haptotactic cue that promotes mechanotransduction [11]. Netrin-1 can work simultaneously as an attractive and a repulsive guidance cue depending on the resulting interaction between the cytoplasmic domains of the dimerized receptors. This cytoplasmic complex will create a platform for the association of downstream signalling effectors. For example, the association of the intracellular P1 domain of DCC and the DB domain of UNC5 results in Netrin-1-induced repulsion [12]. This repulsion is crucial during the early postnatal development of the cerebellum, where it governs the correct extension of parallel fibres from the external granular layer (EGL) neurons and the migration of granule cells to deeper layers of the cerebellum [13]. During postnatal cerebellar development, both UNC5B and UNC5C receptors are expressed in neurons from the EGL. Immediately after birth, postmitotic granule cells descend to the deep EGL and tangentially extend their axons, the parallel fibres. The somata of the granule cells then re-orientate perpendicular to the axons and migrate radially through the molecular layer to form the internal granule layer (IGL) [13]. Netrin-1 secreted by EGL and interneurons of the molecular layer, repel parallel fibres from granule cells, preventing their aberrant growth toward deeper layers of the cerebellum. Thus, understanding the molecular elements involved in the formation of the UNC5 receptor complex is of crucial importance to decipher the mechanisms that govern the specific response of Netrin-1 during brain development.

Transmembrane receptors can be embedded in the plasma membrane within specialized microdomains known as lipid rafts [14]. These microdomains are plasma membrane fractions which are enriched in cholesterol and sphingolipids, thereby conferring a more liquid-ordered phase [15], and are able to selectively include or exclude a wide range of lipid-anchored proteins, as well as transmembrane proteins. Lipid rafts are essential for ensuring the proper signal transduction involved in axon guidance [16] and neural migration [17]. However, although it is known that some UNC5s are localized into rafts [18], the importance of the specific organization of these receptors within raft membrane microdomains during axon guidance remains unknown.

Here, by performing quantitative live-cell microscopy-based techniques such as fluorescence recovery after photobleaching (FRAP) and single-particle tracking photoactivation localization microscopy (sptPALM) in HEK-293AD cells and primary neuronal cultures, we present a full characterization of the membrane distribution of all UNC5(A–D) members. As a consequence, we describe previously unknown properties of different UNC5 members and establish a new classification based on their membrane mobility. Using a combination of pharmacological and genetic approaches in primary neuronal cultures and cerebellar explants, we demonstrate that the integrity of membrane raft microdomains is of crucial importance to translate the UNC5-dependent Netrin-1 response into growth cone collapse and axonal chemorepulsion. These results demonstrate the functional relevance of the organization of UNC5s into microdomains for the development of cerebellar structures.

## Results

### Localization of UNC5(A–D) YFP-tagged receptors

**Fig. 1:**
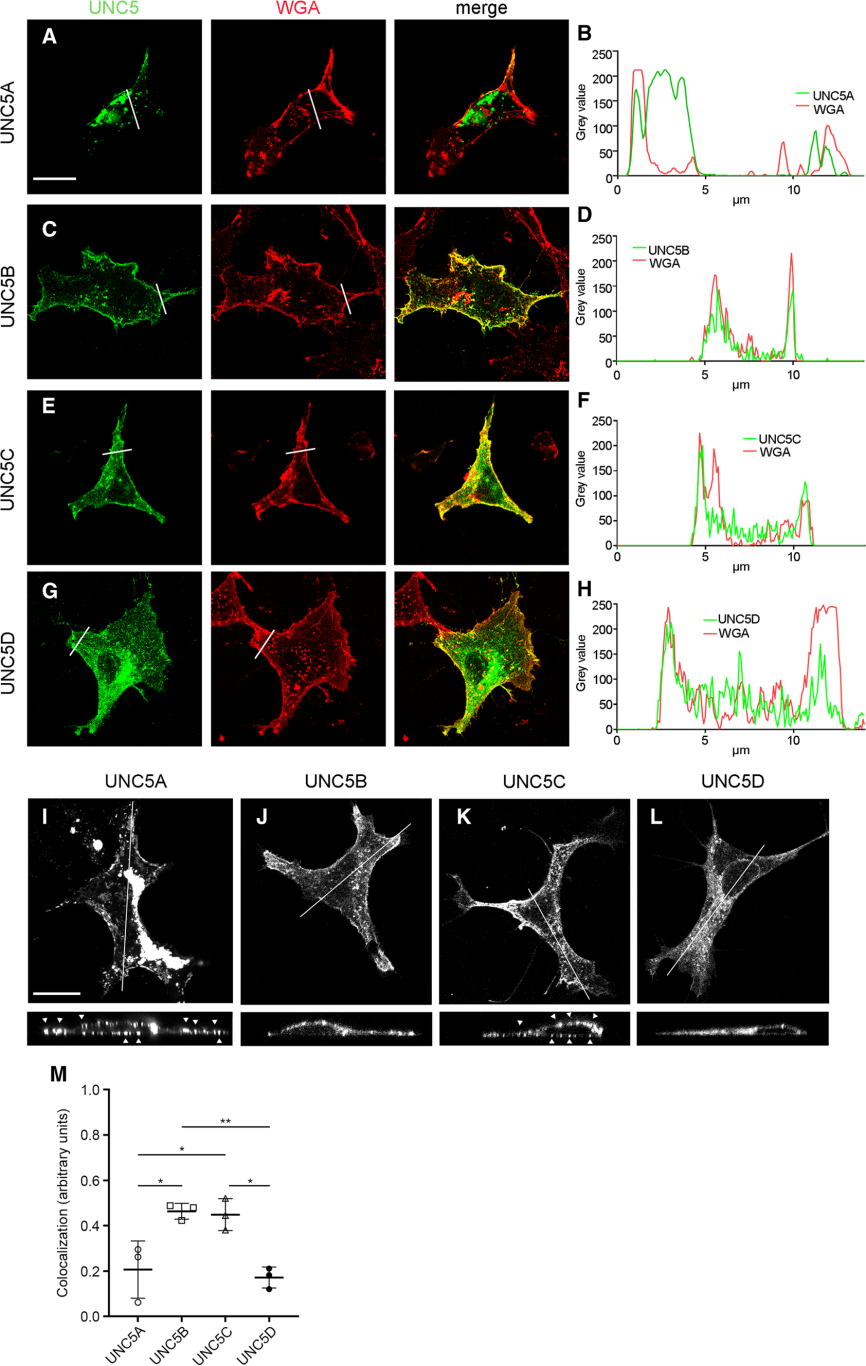
Distribution of YFP-tagged UNC5 receptors expressed in HEK-293AD cells. Representative confocal images of HEK-293AD cells expressing **a** UNC5A-YFP, **c** UNC5B-YFP, **e** UNC5C-YFP and **g** UNC5D-YFP receptors. Scale bar represents 20 µm. **b**, **d**, **f**, **h** Cells were labelled with the plasma membrane marker WGA. Intensities for each channel under the line drawn in each image are plotted in a graphic. **i**-**l** Confocal images from HEK-293AD cells expressing YFP-tagged UNC5A–D receptors. Scale bar represents 20 µm. The lines in each cell illustrate the *x*,*y* axis where the *x*,*z*-representation is obtained. Arrows in **i** and **k** point to clusters of UNC5A and UNC5C, respectively. **m** Quantification of the colocalization observed between the UNC5 receptor channel and WGA channel (range 0–1). Data represent means from measurements of three independent experiments (over 10 cells per experiment) ± SD. One-way ANOVA followed by Tukey's multiple comparison post hoc test comparing all groups; *p ≤ 0.05, **p ≤ 0.01

To investigate the membrane distribution and dynamics of UNC5 receptors involved in the repulsion associated with the recognition of Netrin-1 ligand, we fused the fluorescent protein YFP to the C-terminus of all four members of the UNC5(A–D) family of proteins. We first expressed DNAs encoding each YFP-tagged UNC5 receptor (UNC5-YFP) in HEK-293AD cells and examined their subcellular spatial distribution using confocal microscopy. To determine the proper targeting of the UNC5 receptors to the cell membrane, we specifically labelled cytoplasmic membranes by incubating the cells with a fluorescently tagged wheat germ agglutinin (WGA), allowing us to evaluate the co-distribution of this membrane marker with the UNC5 receptors. We observed that all four UNC5(A–D) constructs colocalized with the WGA cell membrane marker (Fig. 1a, c, e, g). We then plotted the signal intensity of each receptor and the membrane marker along specific regions of cells by drawing a straight region of interest (ROI) which crossed the cell membrane twice and analysed the intensities. As expected, two peaks in the WGA signal were detected in each analysed cell. The two WGA intensity peaks (red), overlapped with peaks (green) corresponding to the UNC5 receptors (Fig. 1b, d, f, h). Although all four YFP-tagged UNC5 constructs traffic to the cell membrane, we observed higher intracellular retention of UNC5A and UNC5D constructs, compared to other UNC5 partners.

To better examine the intracellular distribution of UNC5 receptors, we acquired Z-stacks and composed an *x*-*z* representation of each UNC5 in HEK-293AD cells (Fig. 1i-l). The distribution of all UNC5 receptors along the cell membrane was further verified. Interestingly, we observed a differential pattern of membrane distribution among the receptors. Whereas UNC5A and UNC5C predominantly formed clusters along the cell membrane (Fig. 1i, k, arrows), UNC5B was evenly distributed on the plasma membrane (Fig. 1j). We also verified that UNC5A and UNC5D were the constructs that exhibited more cytoplasmic retention. Interestingly, whereas UNC5A showed partial intracellular accumulation compatible with Golgi-derived structures (Fig. 1i), UNC5D exhibited a distribution more characteristic of the endoplasmic reticulum (Fig. 1l). Analysis of the colocalization with the WGA membrane marker (Fig. 1m) further indicated that UNC5B and UNC5C displayed a predominantly membrane distribution.

The alignment of the aminoacidic sequences of all the UNC5 receptors revealed that the structural domains Ig, TSPI, transmembrane domain and DD are conserved among all four receptors (Supplementary Fig. 1). UNC5A shares 54% and 56% homology with UNC5B and UNC5C, respectively. The homology between UNC5B and UNC5C is 65%, which is the highest homology among all UNC5 members. In contrast, UNC5D is the most distant member in terms of sequence homology. When we analysed the sequence homology of the DDs, UNC5B and UNC5C were revealed to be the closest members, sharing 67% homology. The DD of UNC5A shares 49% homology with UNC5B and 48% with UNC5C. UNC5D shares 43% homology with UNC5A, and 55% and 50% homology with UNC5B and UNC5C, respectively. Therefore, unlike the comparison of the whole sequence of UNC5 members, in this instance, UNC5A proved to be the most distant family member.

### Differential lateral mobility of UNC5(A–D) receptors in the plasma membrane

**Fig. 2:**
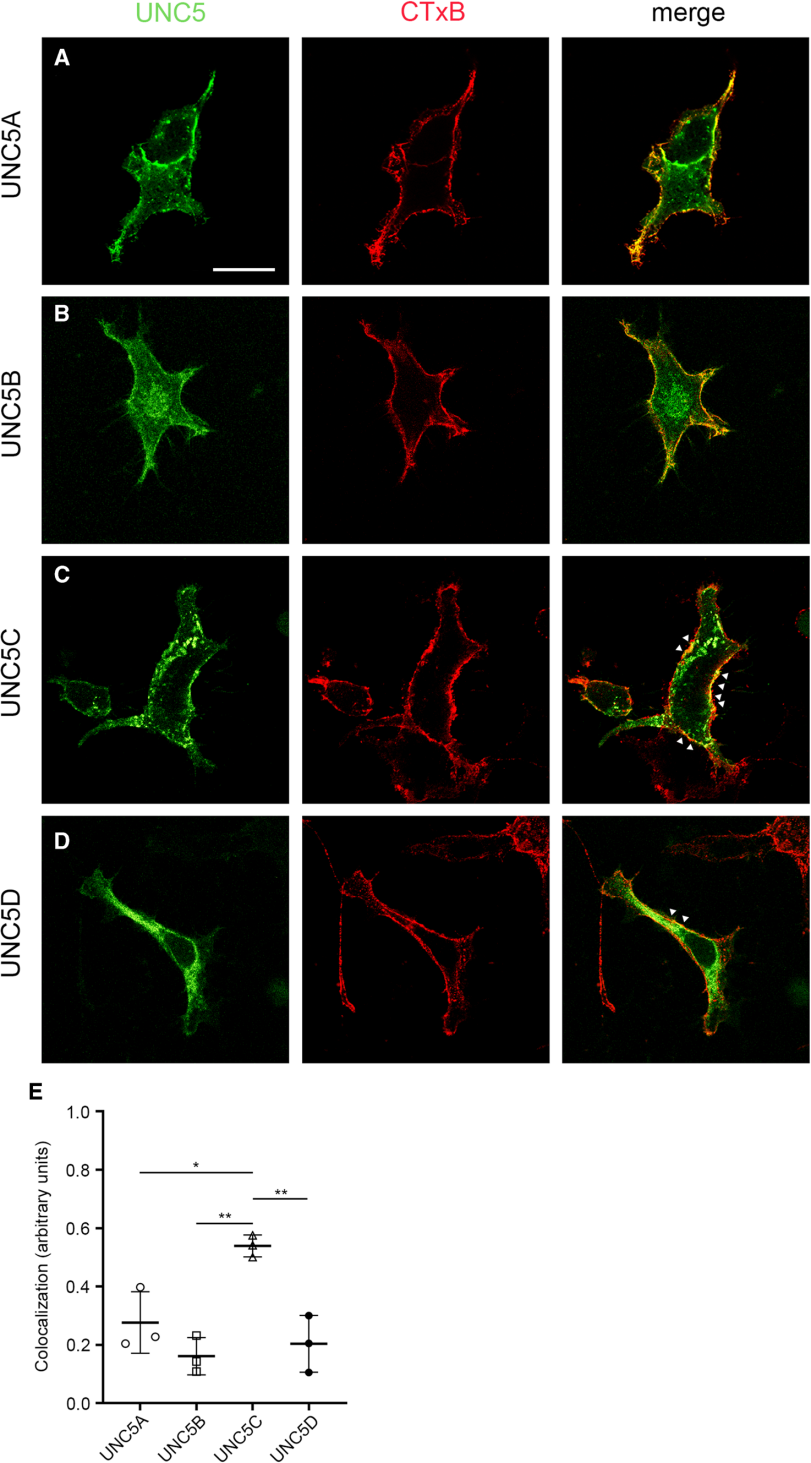
Colocalization of UNC5(A–D)-YFP receptors with the lipid raft membrane marker CTxB. Representative confocal images of HEK-293AD cells expressing **a** UNC5A-YFP, **b** UNC5B-YFP, **c** UNC5C-YFP and **d** UNC5D-YFP receptors. Cells were labelled with CTxB, a lipid raft marker in the plasma membrane. Arrowheads in **c** indicate the presence of clusters of UNC5C. Scale bar represents 20 µm. **e** Quantification of the colocalization observed between the UNC5 receptors channel and CTxB channel (range 0–1). Data represent means from measurements of three independent experiments (over 10 cells per experiment) ± SD. One-way ANOVA followed by Tukey's multiple comparison post hoc test comparing all groups; *p ≤ 0.05, **p ≤ 0.01

**Fig. 3:**
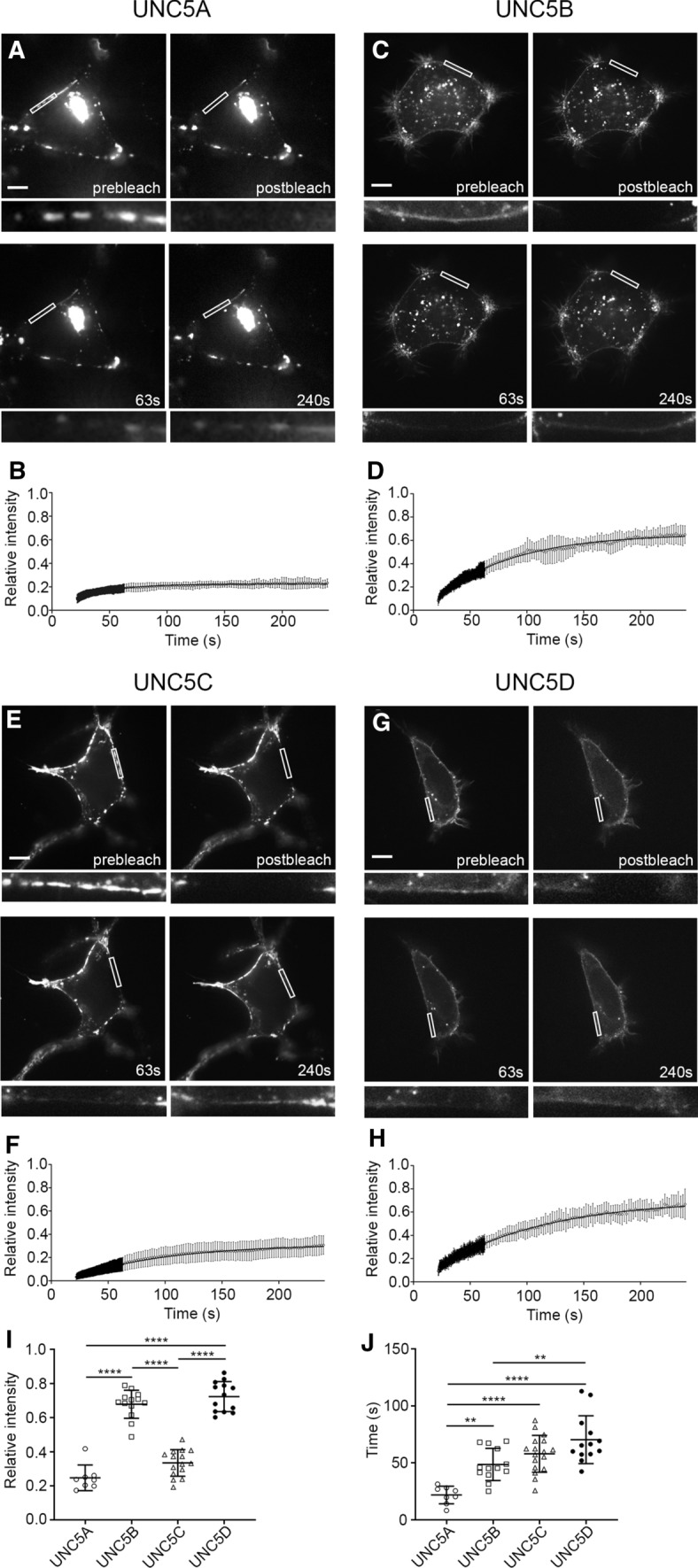
Differences in the FRAP lateral mobility of UNC5(A–D)-YFP receptors. Representative images from FRAP experiments at different time points (prebleach, 0 s postbleach, and 63 s and 240 s postbleach) of cells expressing **a** UNC5A-YFP, **c** UNC5B-YFP, **d** UNC5C-YFP or **f** UNC5D-YFP. The boxed area within each cell indicates the region where the fluorescence signal was photobleached and the recovery was analysed. Graphs representing the average recovery after photobleaching of **b** UNC5A-YFP, **d** UNC5B-YFP, **f** UNC5C-YFP or **h** UNC5D-YFP. Data are displayed as mean ± SD. *n* = 8 cells in **b**, *n* = 13 cells in **d**, *n* = 15 cells in **f** and *n* = 13 cells in **h**. Scale bar represents 20 µm. **i** Plotting and comparison of the Mf calculated for each of the UNC5 receptors. **j** Plotting and comparison of the calculated t_1/2_ for each of the UNC5 receptors. One-way ANOVA followed by Tukey's multiple comparison post hoc test comparing all groups was used in **i** and **j**; **p ≤ 0.01, ****p ≤ 0.0001

Most UNC5 receptors are organized within raft microdomains [18]. Here, we first wanted to verify whether all UNC5 members are localized into cholesterol-enriched lipid rafts. Based on immunocytochemical analysis of the colocalization of membrane UNC5 receptors with the raft marker cholera toxin B-subunit [19] (CTxB-Alexa Fluor 555) we found that all four members of the UNC5 family partially colocalize with lipid raft membrane regions (Fig. 2a–d), with UNC5C exhibiting the greatest colocalization (Fig. 2e). To quantify the retention of UNC5 receptors in specific membrane microdomains, we performed FRAP assays, elucidating different membrane mobility dynamics for each receptor (Fig. 3). In HEK-293AD cells expressing each of the UNC5(A–D) receptors, a small ROI along the cell surface was bleached using the 488 nm laser line. The recovery of fluorescence within the bleached area was monitored by acquiring images in the prebleaching, bleaching and recovery phases (63 s and 240 s). Fluorescence values were normalized to the prebleach intensity and corrected with the background intensity and the cumulative unspecific photobleaching due to the scanning protocol. When we analysed the membrane dynamics of the receptors, UNC5A showed the slowest fluorescence recovery rate, with an average mobile fraction (Mf) of 0.22 ± 0.003 (Fig. 3a, b). UNC5B had a Mf of 0.65 ± 0.007 (Fig. 3c, d), while UNC5C, which together with UNC5A exhibited a marked clustered distribution, had a Mf of 0.31 ± 0.0081 (Fig. 3e, f). UNC5D exhibited the fastest fluorescence recovery rate, with a Mf of 0.7 ± 0.012 (Fig. 3g, h). Overall, comparisons between the Mf of the UNC5 receptors revealed that UNC5B and UNC5D receptors exhibited a higher ratio of mobile molecules, whereas UNC5A and UNC5C receptors exhibited a significantly lower Mf (Fig. 3i). The analysis of halftimes (t_1/2_) revealed that UNC5A was the receptor with the lowest t_1/2_ (21.86 s), UNC5D the highest t_1/2_ (70.43 s) and UNC5B and UNC5C exhibited intermediate values (48.69 s and 58.02 s, respectively). As a comparison, we also analysed the membrane dynamics of the Netrin-1 receptor DCC. Its Mf was 0.59 ± 0.007, similar to the value obtained for UNC5B (Supplementary Fig. 2a). Interestingly, whereas all UNC5 receptors localize in lipid raft domains, our results using FRAP analysis suggest that the two receptors (UNC5A and UNC5C) that distribute in distinct surface puncta along cell membranes share a similar low lateral membrane mobility. Receptors (UNC5B and UNC5D) which are evenly distributed in the cell membrane exhibit a faster lateral mobility.

**Fig. 4:**
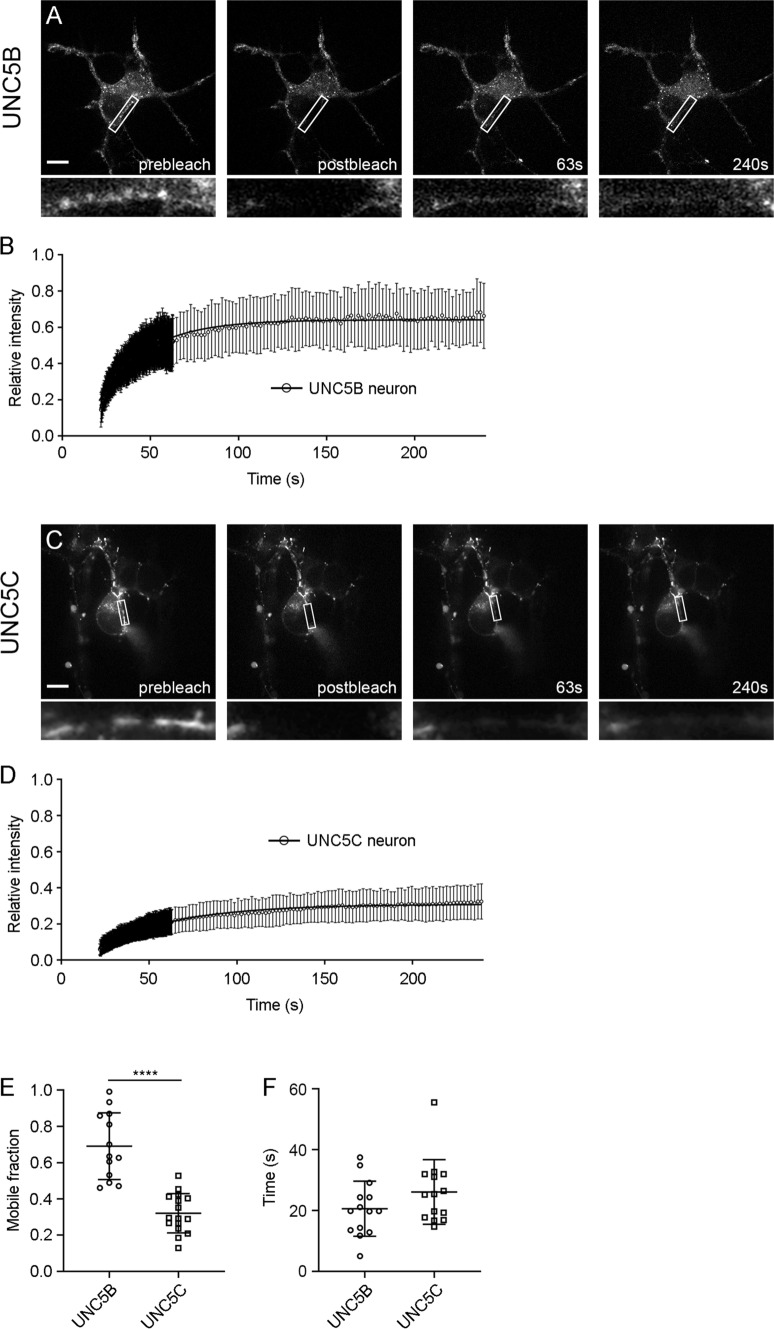
FRAP lateral mobility of UNC5B-YFP and UNC5C-YFP in transfected hippocampal neurons. **a** Representative images from FRAP experiments at different time points for a UNC5B-YFP-transfected hippocampal neuron (prebleach, 0 s postbleach, and 63 s and 240 s postbleach). Scale bar represents 20 µm. **b** Average recovery after photobleaching of UNC5B-YFP expressed in hippocampal neurons. **c** Representative images from FRAP experiments at different time points for a UNC5C-YFP-transfected hippocampal neuron. Scale bar represents 20 µm. **d** Average recovery after photobleaching of UNC5B-YFP expressed in hippocampal neurons. FRAP curve data are displayed as mean ± SD. *n* = 13 neurons in **b** and *n* = 16 neurons in **d**.** e** Comparison of the Mf of UNC5B-YFP and UNC5C-YFP in the soma of hippocampal neurons. **f** Comparison of the t_1/2_ of UNC5B-YFP and UNC5C-YFP expressed in hippocampal neurons. Data are displayed as mean ± SD. An unpaired two-tailed Student’s *t* test was used in **e** and **f**; ****p ≤ 0.0001

Our results reveal subtle differences in the membrane organization of the four members of the UNC5 family. However, the role of the different UNC5 receptors during axonal guidance processes have been most widely characterized for UNC5B and UNC5C. Both receptors participate in the correct elongation of EGL axons during the development of the cerebellum [13], a well-established model of axonal chemorepulsion, and are responsible for the repulsion by Netrin-1 observed in this neuronal system. They control the correct extension of EGL parallel fibres, preventing their aberrant growth toward deeper layers of the cerebellum [13]. To better determine whether the mobility ratios found in HEK-293 cells are consistent with the membrane dynamics of the receptors in neurons, we performed FRAP experiments in hippocampal neurons transfected with UNC5B and UNC5C constructs (Fig. 4). As EGL cerebellar granule cells are one of the smallest neuron of all types in the brain, a proper FRAP analysis of transfected UNC5 receptors was not possible in these cells. However, using the same FRAP protocol as in HEK-293AD cell experiments, we were able to demonstrate that the Mf and t_1/2_ for UNC5B (Fig. 4a, b, e, Mf = 0.64 ± 0.009, Fig. 4f, t_1/2_ = 20.57 s) and UNC5C (Fig. 4c, d, e, Mf = 0.3 ± 0.005, Fig. 4f, t_1/2_ = 26.09 s) in the soma of hippocampal neurons exhibited the same behaviour as in HEK-293AD cells, with different M_f_ but similar t_1/2_. The Mf exhibited by the DCC receptor in hippocampal neurons was 0.4 ± 0.01, and the t_1/2_ was 36.99 s, both values being lower than those obtained in HEK-293AD cells (Supplementary Fig. 2).

### Membrane dynamics of UNC5B and UNC5C are modified after cholesterol depletion and death domain (DD) deletion

**Fig. 5:**
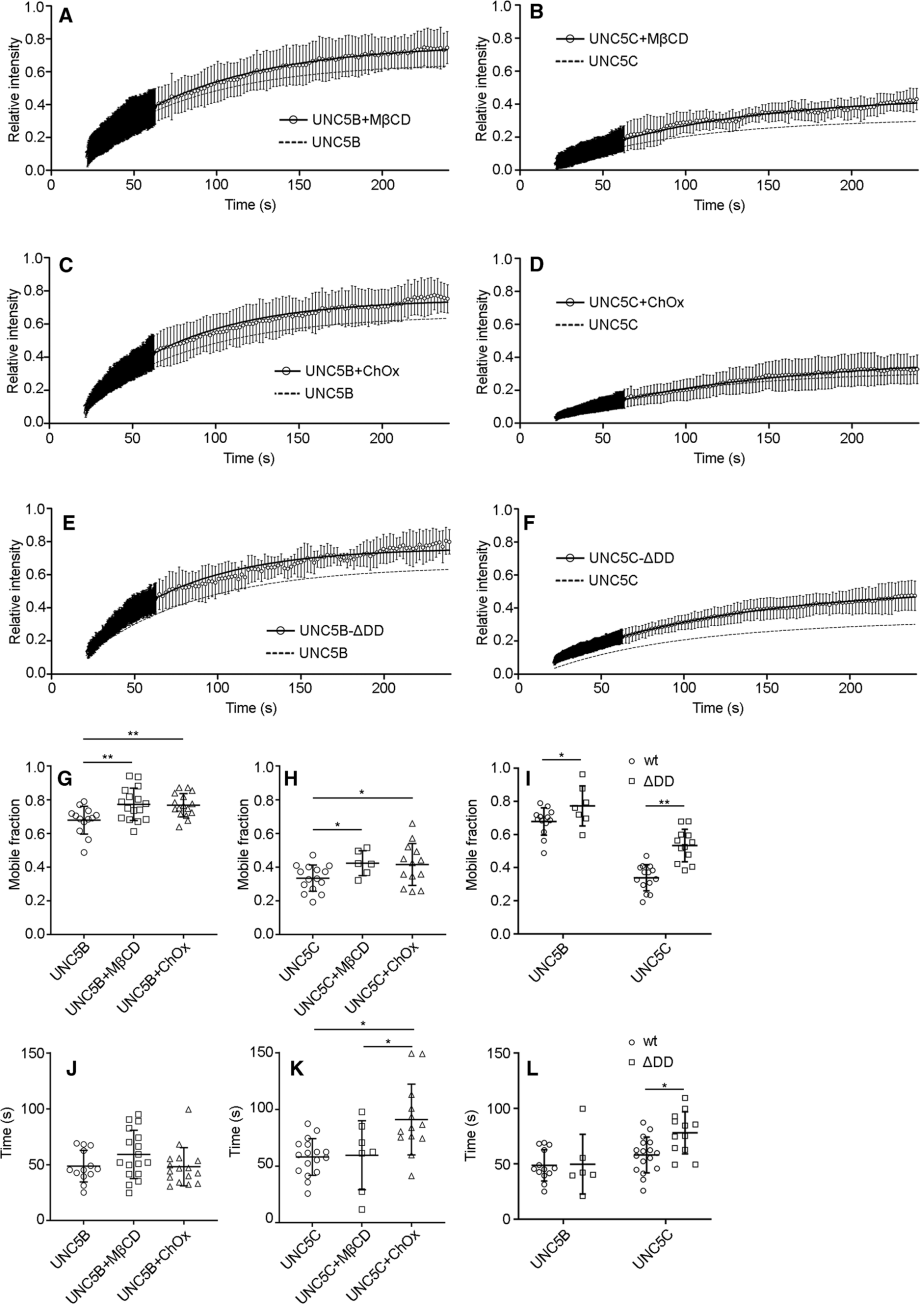
Membrane dynamics of UNC5B and UNC5C after cholesterol depletion and DD deletion. Average recovery after photobleaching of **a**, **c** UNC5B-YFP or **b**, **d** UNC5C-YFP expressed in HEK-293AD cells treated with **a**, **b** MβCD (1 mM, 10 min) or **c**, **d** ChOx (2U, 2 h). (E, F) Average recovery after photobleaching of **e** UNC5B-ΔDD-YFP and **f** UNC5C-ΔDD-YFP. For comparison, the dashed line in each plot represents the dynamics of each receptor under control conditions. All curves were fitted to a single exponential. **g** Comparison of the Mf of UNC5B-YFP under each treatment or **h** UNC5C-YFP under each treatment. **i** Comparison of the Mf of full-length receptors with those exhibited by truncated receptors, lacking the DD. Data are displayed as mean ± SD. *n* = 16 cells in **a**, *n* = 16 cells in **b**, *n* = 6 cells in **c**,, *n* = 13 cells in **d**, *n* = 7 cells in **e** and *n* = 12 cells in **f**. One-way ANOVA followed by Tukey's multiple comparison post hoc test was used to compare the Mf of all groups with the vehicle-treated group in **g** and **h**; An unpaired two-tailed Student’s *t* test was used in **i**. One-way ANOVA followed by Tukey's multiple comparison post hoc test was used to compare the Mf of all groups with the vehicle-treated group in **j** and **k**; An unpaired two-tailed Student’s *t* test was used in **l**; *p ≤ 0.05, **p ≤ 0.01

Our results suggest that UNC5 receptors, in particular UNC5B and UNC5C, are localized in cholesterol-enriched lipid raft membrane microdomains. To evaluate the importance of cholesterol-enriched structures on receptor membrane dynamics, we first performed classical biochemical fractionation through sucrose gradient centrifugation and protein co-sedimentation with detergent-resistant membranes (DRM) or detergent-soluble membranes (DSM). DRM are known as lipid raft fractions and are formed by proteins that cannot be solubilized at low temperatures with non-ionic detergents, such as caveolin and flotillin, among many others. DSM are the non-raft fractions, representing proteins that can be solubilized with non-ionic detergents, such as clathrin and the transferrin receptor. We depleted membrane cholesterol using methyl-β-cyclodextrin (MβCD) [20], which removes membrane cholesterol by forming inclusion complexes, thereby resulting in the dissociation of raft structures. Both UNC5B and UNC5C partially co-distributed with the lipid raft protein caveolin in a MβCD- sensitive manner (Supplementary Fig. 3). We then performed FRAP experiments on UNC5B and UNC5C after disruption of lipid rafts using different types of cholesterol-depleting reagents, MβCD and cholesterol oxidase (ChOx). ChOx is an enzyme that catalyses the oxidation and isomerization of cholesterol into oxysterol, thus reducing the total level of cholesterol in the cells [21]. UNC5B exhibited an increment of its Mf after treatment with MβCD (Mf = 0.75 ± 0.008) (Fig. 5a, g) or ChOx (Mf = 0.77 ± 0.013) (Fig. 5c, g). Similarly, UNC5C showed increased lateral mobility after treatment with MβCD (Mf = 0.45 ± 0.017) (Fig. 5b, h) or ChOx (Mf = 0.39 ± 0.016) (Fig. 5d, h). The evaluation of t_1/2_ showed that this parameter was only affected in UNC5C (Fig. 5j, k). Taken together, these data suggest that depletion of cell membrane cholesterol with MβCD or ChOx increases the amount of UNC5B and UNC5C that can freely diffuse within the membrane.

It has been reported by biochemical fractionation that the DD of UNC5B is necessary for its specific localization to lipid rafts [18]. We, therefore, next investigated whether deletion of the DD in UNC5B and UNC5C affects the membrane dynamics of these receptors. We expressed YFP-tagged truncated forms of UNC5B and UNC5C receptors lacking the DD (UNC5B-ΔDD and UNC5C-ΔDD, respectively) in HEK-293AD cells, and performed FRAP experiments to assess the dynamics of both truncated forms (Fig. 5e, f). Deletion of the DD increased the Mf of both receptors: Mf = 0.76 ± 0.01 for UNC5B-ΔDD; Mf = 0.52 ± 0.011 for UNC5C-ΔDD. This increment was higher for UNC5C-ΔDD (40.3%) than for UNC5B-ΔDD (14.4%) (Fig. 5i). Similar to the results obtained using MβCD, t_1/2_ was only affected for UNC5C (Fig. 5l). These results suggest that the DD is a key element for UNC5 receptors to maintain their biophysical properties at the cell membrane.

### UNC5C is organized in stable membrane clusters

**Fig. 6:**
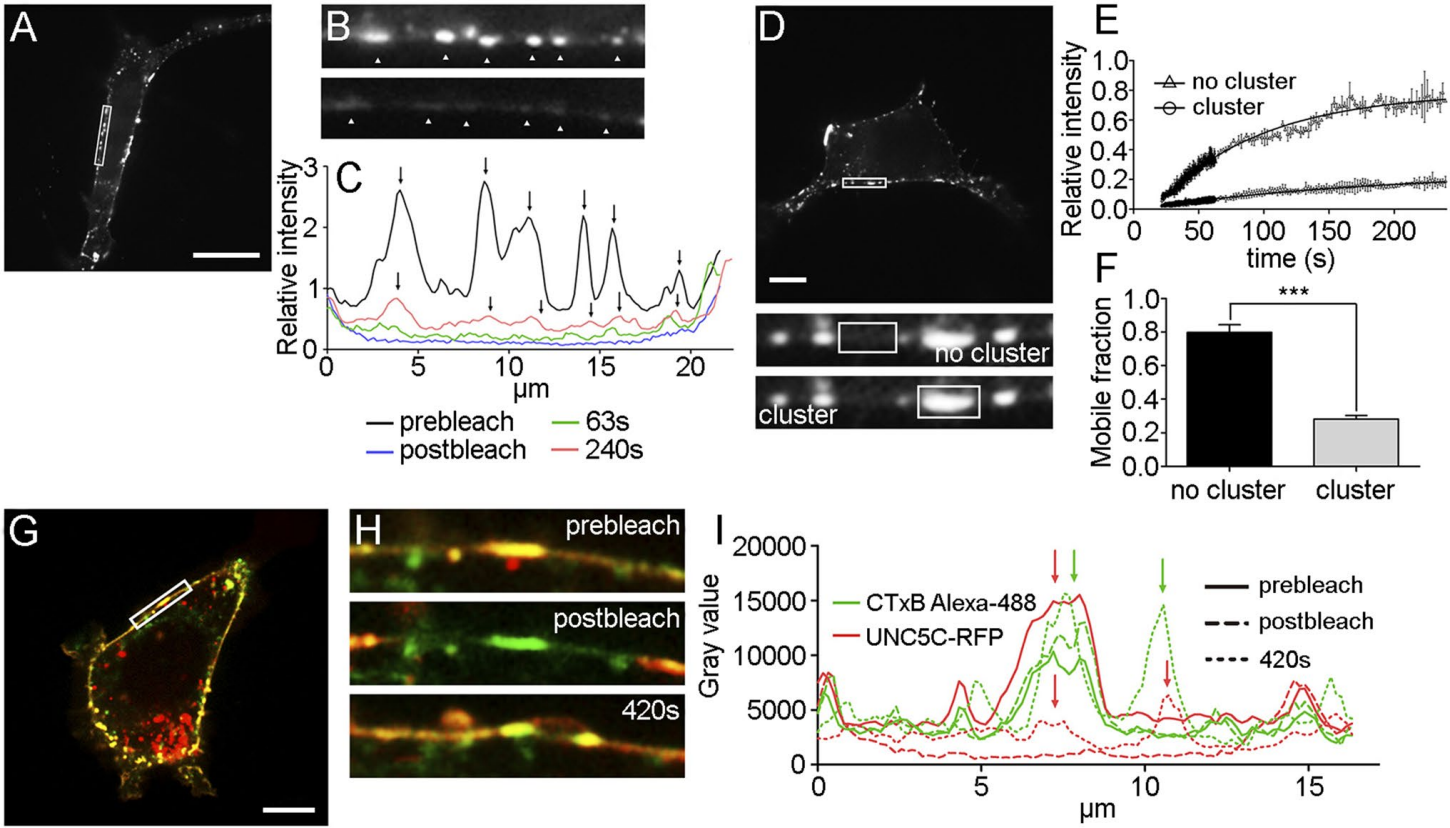
Organization of UNC5C into stable membrane clusters. **a** Representative image of a HEK-293AD cell expressing UNC5C-YFP, and the photobleached area (white ROI) used for the FRAP analysis. Scale bar represents 20 µm. **b** Magnification of the photobleached area showing two different time points of the FRAP experiment, prebleach and 240 s postbleach. Arrowheads indicate UNC5C clusters along the plasma membrane. **c** Representation of UNC5C-YFP relative intensities within the bleached region and at different time points of the FRAP experiment. Arrows indicate the localization of initial UNC5C clusters (prebleach) and after 240 s postbleach. **d** HEK-293AD cell expressing UNC5C-YFP and the photobleached area (white ROI) used for the FRAP analysis. Scale bar represents 10 µm. Magnification of the photobleached area indicating the two types of regions (white ROIs) used to perform FRAP analysis, inside (“cluster”) and outside (“no cluster”) of cluster areas. **e** Average recovery after photobleaching of UNC5C-YFP obtained from “cluster” or “no cluster” areas. **f** Comparison of the Mf of UNC5C-YFP in “cluster” and “no cluster” regions. **g** HEK-293AD cell labelled with CTxB (green) and expressing UNC5C-RFP. Scale bar represents 10 µm. The ROI indicates where the photobleaching was performed for the FRAP analysis. **h** Magnification of the photobleached area showing three different time points of the FRAP experiment, prebleach, 0 s postbleach and 240 s postbleach. **i** Representation of UNC5C-RFP relative intensities within the bleached region and at different time points of the FRAP experiment. Arrows indicate the localization of UNC5C clusters (red arrow) and CTxB (green arrow) at different time points. An unpaired two-tailed Student’s *t* test was used in **f**. ***p ≤ 0.001. All scale bar described above represent 20 µm

Protein clustering is used by neuronal proteins to facilitate the formation of complexes that transduce their downstream signalling more efficiently. When comparing UNC5B and UNC5C, the results described above demonstrate that only the later forms distinguishable membrane clusters. To examine the stability of individual UNC5C clusters located along the plasma membrane, we performed the same FRAP protocol as previously outlined. We photobleached an area of the membrane containing clustered and non-clustered receptors (Fig. 6a) and measured the fluorescence recovery at four different time points, prebleach (immediately before the photobleaching), postbleach (0 s, immediately after the photobleaching), and 63 s and 240 s after photobleaching (Fig. 6b, c). The fluorescence intensities were normalized to their prebleach values and plotted against their specific localizations (Fig. 6c). The six intensity peaks plotted (arrows in Fig. 6c) corresponded to six clusters of UNC5C located along the plasma membrane (arrowheads in Fig. 6b). Fluorescence recovery 62 s after photobleaching was indistinguishable from the postbleach instant. However, 240 s after photobleaching, the intensities of all six UNC5C signals had partially recovered in the same spatial position as the original photobleached clusters. Non-bleached molecules can move in and out of clustered regions. As UNC5C was unevenly distributed across the bleached ROI, we measured its recovery by selecting smaller sub-ROIs containing exclusively bleached clusters (Fig. 6d “cluster”) or bleached non-clusters (Fig. 6d “non-cluster”). Strikingly, the Mf of clustered proteins was 0.25 ± 0.025, while the Mf in a non-clustered region was 0.79 ± 0.023 (Fig. 6e, f). These results suggest that UNC5C shows two distinguishable dynamics along the cell membrane, immobilized and freely diffusive.

We then investigated whether these clusters were enriched in the lipid raft marker CTxB-488. UNC5C-RFP was transfected into HEK-293AD cells and incubated with CTxB. UNC5C cluster regions colocalized with CTxB labelling, suggesting that receptor clusters occupy lipid raft microdomains (Fig. 6g). Next, we performed FRAP on a clustered area using a 568 laser beam to specifically photobleach UNC5C receptors without affecting the raft marker (Fig. 6h). After 420 s of photobleaching, UNC5C preferentially redistributed back and reconstituted its clusters within pre-existing cholesterol-enriched regions (Fig. 6i). These results reinforce the idea that UNC5C receptors accumulate into membrane raft microdomains, forming a temporally stable structure.

### UNC5B and UNC5C receptors have a differential nanoscale organization

SptPALM is a super-resolution microscopy technique that allows investigation of the mobility of single molecules in vitro and in vivo [22, 23]. We tagged UNC5B and UNC5C receptors with mEos2, a photoswitchable fluorescent protein that, upon stochastic photoconversion, allows imaging of single-molecule trajectories with a resolution below the diffraction limits of light. UNC5B and UNC5C located in the plasma membrane were imaged using total internal reflection fluorescence (TIRF) microscopy. Analysis of thousands of single-molecule trajectories (˃ 1000 trajectories per cell, 12–15 cells) allowed quantification of their mean square displacement (MSD), the distribution of obtained diffusion coefficients and the frequency of different mobility types (trapped, confined and free).

**Fig. 7:**
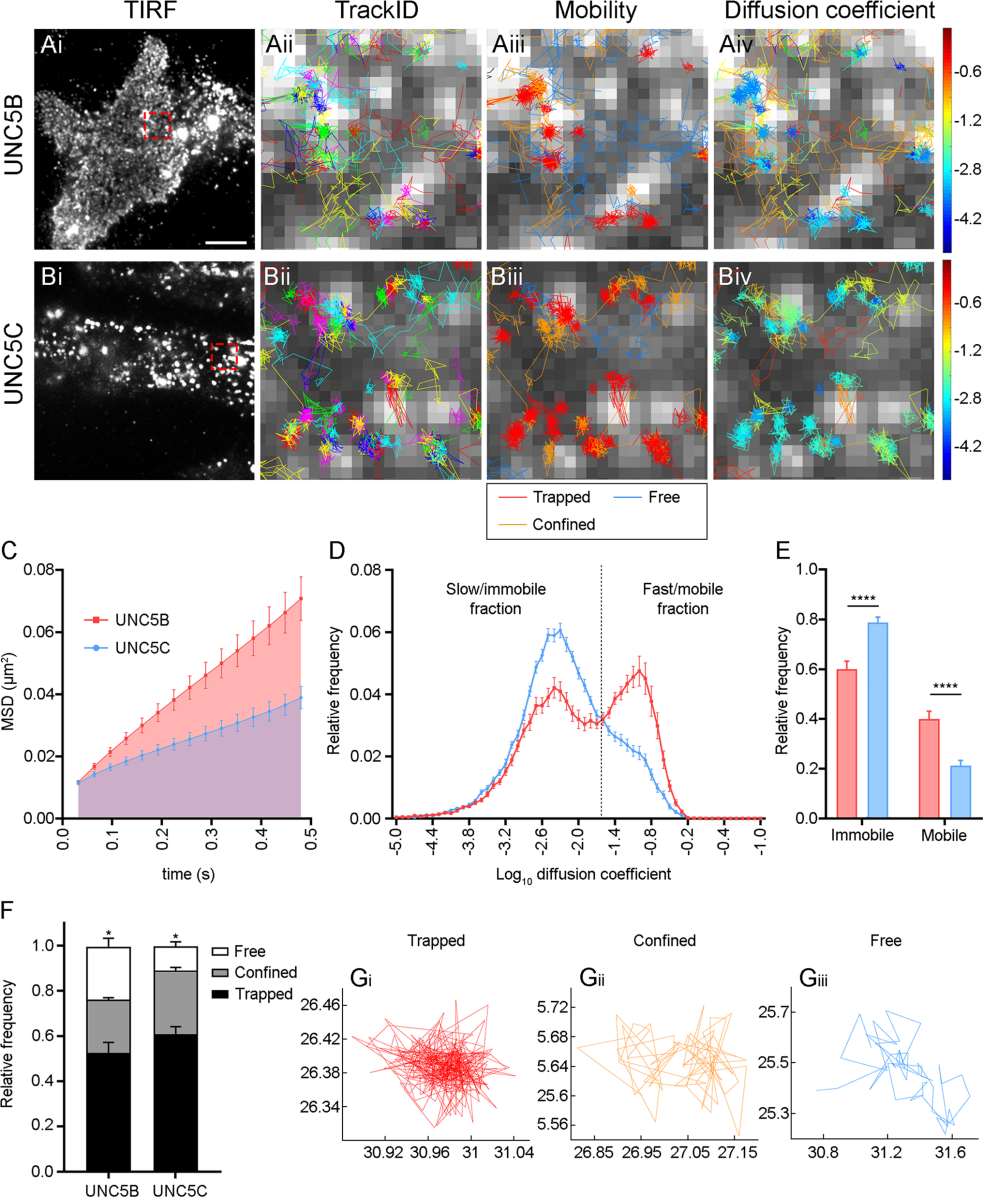
sptPALM reveals new insights into UNC5B and UNC5C mobility and diffusion constants. **a**, **b** Representative epifluorescence TIRF images of HEK-293AD cells expressing **ai** UNC5B-mEos2 or **bi** UNC5C-mEos2, acquired before photoconversion of mEos2 molecules. Scale bar represents 30 µm. Insets in **ai** and **bi** are shown at a higher magnification. Individual trajectories with arbitrary colours for **aii** UNC5B and **bii** UNC5C receptors are superimposed on an image of single mEos2 molecules converted to red form to illustrate the density of tracked molecules. Classification of the trajectories into trapped (red), confined (orange) or free (blue) for **aiii** UNC5B and **biii** UNC5C receptors was done using moment scaling spectrum analysis. Classification of the trajectories for **aiv** UNC5B and **biv** UNC5C receptors based on their diffusion coefficients. Trajectories with low diffusion coefficients are shown in cold colours and trajectories with high diffusion coefficients are shown in warm colours. **c** Average mean square displacement (MSD) as a function of time for UNC5B (blue) and UNC5C (red) receptors. **d** Distribution of the diffusion coefficients shown in a semi-log plot. The threshold to distinguish the immobile (Log10[D] ≤ − 1.6) and mobile (Log10[D] > − 1.6) fraction of molecules is indicated with a dashed line. **e** Comparison of the immobile and mobile populations calculated from the relative frequencies for UNC5B (blue) and UNC5C (red) receptors. **f** Comparison of relative frequencies of the different types of sptPALM trajectories described for UNC5B and UNC5C receptors (trapped, confined and free). **g** Representative examples of trajectories based on their diffusion coefficients and the occupied radius of single molecules, classified as **gi** trapped, **gii** confined and **giii** free trajectories. Data are displayed as mean ± SEM. *n* = 12—15 cells from 4 independent experiments. An *χ*^2^ test was performed to evaluate the distribution of the results into different categories in **e** and **f**, obtaining a *p*-value < 0.002 and < 0.0001, respectively. An unpaired two-tailed Student’s *t* test with the Holm-Šídák correction for multiple comparisons was used. *p = 0.0210, ****p ≤ 0.0001

UNC5B-mEos2 and UNC5C-mEos2 were transfected into HEK-293AD cells. We first imaged cells with a 488 nm laser line to detect transfected cells and obtain a low-resolution TIRF image of UNC5 receptors distributed along the cell membrane immediately adjacent to the glass surface of the dish (Fig. 7ai, bi). We then used a low power 405 nm laser line to stochastically photoconvert a small number of mEos2 molecules from green-emitting to red-emitting. Simultaneously, we used a high power 561 nm laser line to excite the red photoconverted molecules, acquiring their signals until they had photobleached. This allowed us to visualize and track spatially separated single UNC5B-mEos2 and UNC5C-mEos2 proteins. Analysis of single-molecule trajectories from UNC5B and UNC5C receptors (Fig. 7aii, bii) revealed their heterogeneous mobility and diffusion coefficients (Fig. 7aiii, aiv, biii, biv), identifying populations of trapped, confined and freely mobile receptors. Comparison of the MSD curves showed that UNC5B molecules are more mobile than those of UNC5C (Fig. 7c), with an area under the MSD curve of 0.01875 ± 0.002 µm^2^ s for UNC5B, and 0.01143 ± 0.001 µm^2^ s for UNC5C. The distribution of diffusion coefficients showed two distinguishable populations for UNC5B and UNC5C receptors, an immobile population and a mobile population (Fig. 7d). When compared, the immobile and mobile fractions for both receptors were significantly different. The UNC5C receptor had a higher immobile population (Fig. 7e). Based on the diffusion coefficients and the area swept by the molecule, each trajectory could be classified into one of three different categories: trapped (Fig. 7gi), confined (Fig. 7gii) or free (Fig. 7giii). UNC5C exhibited more trapped trajectories and very few free ones when compared with UNC5B. Nonetheless, the proportions of confined trajectories were similar for both receptors (Fig. 7f).

### Cholesterol removal blocks Netrin-1-dependent axonal collapse and repulsion in EGL neurons.

**Fig. 8:**
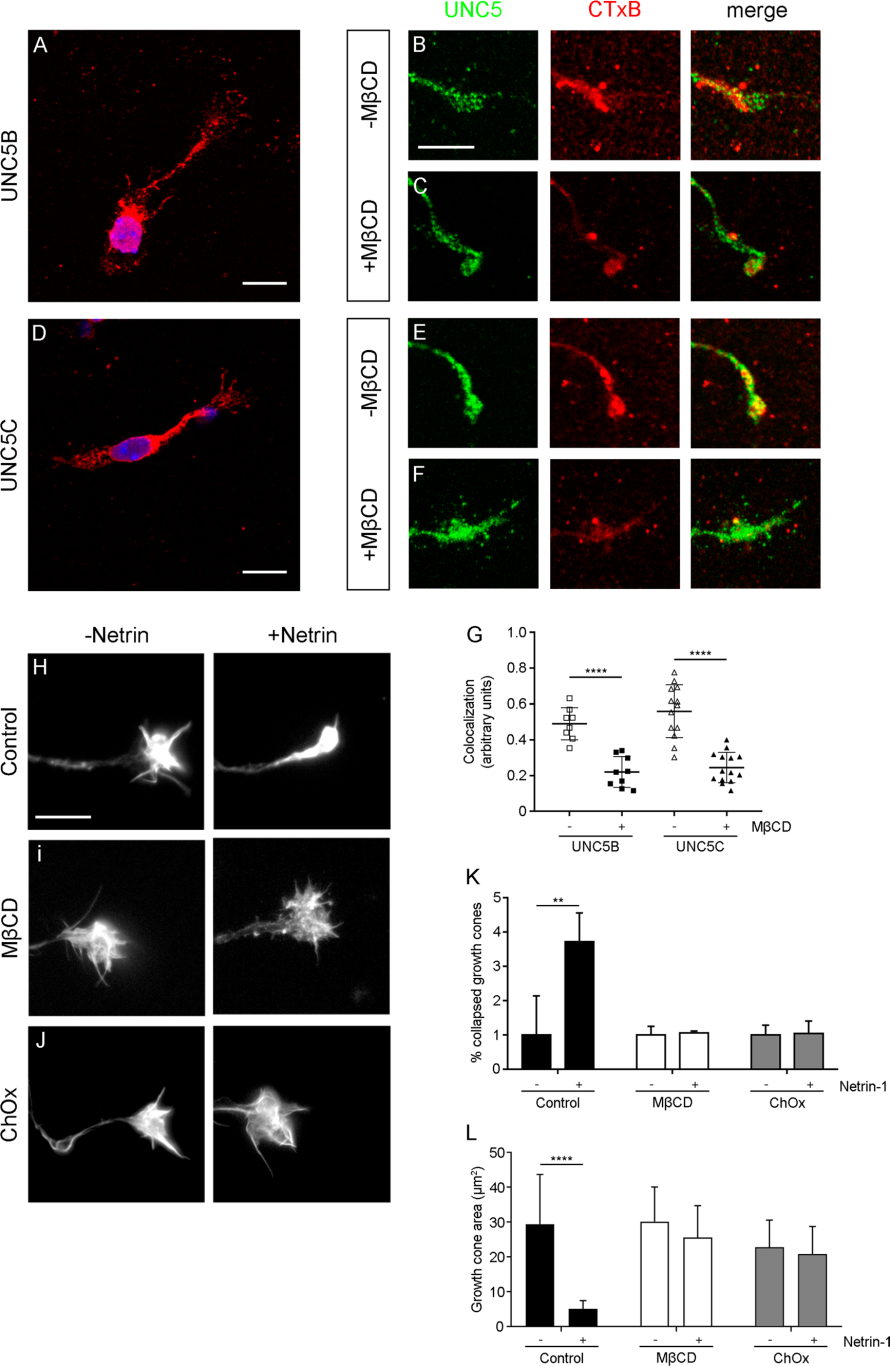
Effect of depleting membrane cholesterol on the Netrin-1-induced collapse of EGL neurons. **a**, **d** Representative confocal images of EGL neurons from P4 mice, immunostained against UNC5B or UNC5C. Scale bar represents 10 µm. **b**, **c** Colocalization of UNC5B with the raft marker CTxB after treating the neurons with **b** control medium or **c** MβCD (1 mM, 10 min). Scale bar represents 15 µm. **e**, **f** Colocalization of UNC5C with the raft marker CTxB after treating the neurons with **e** control medium or **f** MβCD (1 mM, 10 min). **g** Quantification of the colocalization observed between the UNC5B and UNC5C receptor channels and the CTxB channels (range 0–1). Data are represented as means ± SD, n ranges from 8 to 14 growth cones analysed. Two-way ANOVA followed by Tukey's multiple comparison post hoc test was used for the statistical analysis. **h**-**j** Representative confocal images of growth cones from EGL neurons treated with either control medium or Netrin-1-supplemented medium (300 ng/mL, 45 min). Prior to Netrin-1 treatment, cells were treated with **h** control medium, **i** MβCD (1 mM, 10 min) or **j** CO (2U, 2 h). **k** The percentage of collapsed growth cones was calculated and plotted in a graph for each treatment. **l** Growth cone area was measured and plotted in a graph for each treatment. Data represent mean ± SD. The unpaired two-tailed Student’s *t* test was used. **p ≤ 0.01, ****p ≤ 0.0001. All scale bar described above represent 10 µm

The role of the UNC5/Netrin-1 has been mainly studied in relation to the organization of EGL neurons during early postnatal development of the cerebellum, controlling Netrin-1-mediated chemorepulsion of growing parallel fibres [13, 24, 25]. These types of neurons co-express DCC, neogenin, DSCAM, UNC5B and UNC5C that sense Netrin-1 expressed in the EGL and interneurons of the molecular layer of the cerebellum [13]. We isolated mouse EGL neurons from postnatal mice at day 4 (P4), grew them over 3 days in vitro (3DIV) and performed immunostaining against UNC5B and UNC5C receptors, to detect their presence within growth cones (Fig. 8a, b). To investigate whether the differential distribution of UNC5B and UNC5C found in transfected HEK-293AD cells and hippocampal neurons was also observed in EGL neurons, we used the raft marker CTxB, revealing that both endogenous receptors partially colocalize with CTxB (Fig. 8b, e, g). However, this colocalization disappeared when the neurons were incubated with MβCD (1 mM, 10 min) (Fig. 8c, f, g).

Having demonstrated the distribution of UNC5 receptors into specific raft microdomains in EGL neurons, we next wanted to evaluate the functional relevance of this membrane organization for Netrin-1-dependent neuronal chemorepulsion. EGL neurons at 3DIV were treated with MβCD (1 mM, 10 min) or CO (2U, 2 h) to dissociate the raft microdomains before inducing growth cone repulsion and collapse by incubation with Netrin-1 (300 ng/mL) for 45 min. Phalloidin-TRITC was used to stain growth cone actin cytoskeleton, providing *bona fide* information of growth the cone organization. Collapsed growth cones, recognized based on their typical shrivelled, pencil-like shape, were counted. Our results revealed that, whereas Netrin-1 increases growth cone collapse in EGL neurons (Fig. 8h, k, l), this process is inhibited after preincubation with lipid raft-disrupting agents (Fig. 8i-l), suggesting that cholesterol levels are important for Netrin-1-mediated growth cone collapse.

**Fig. 9:**
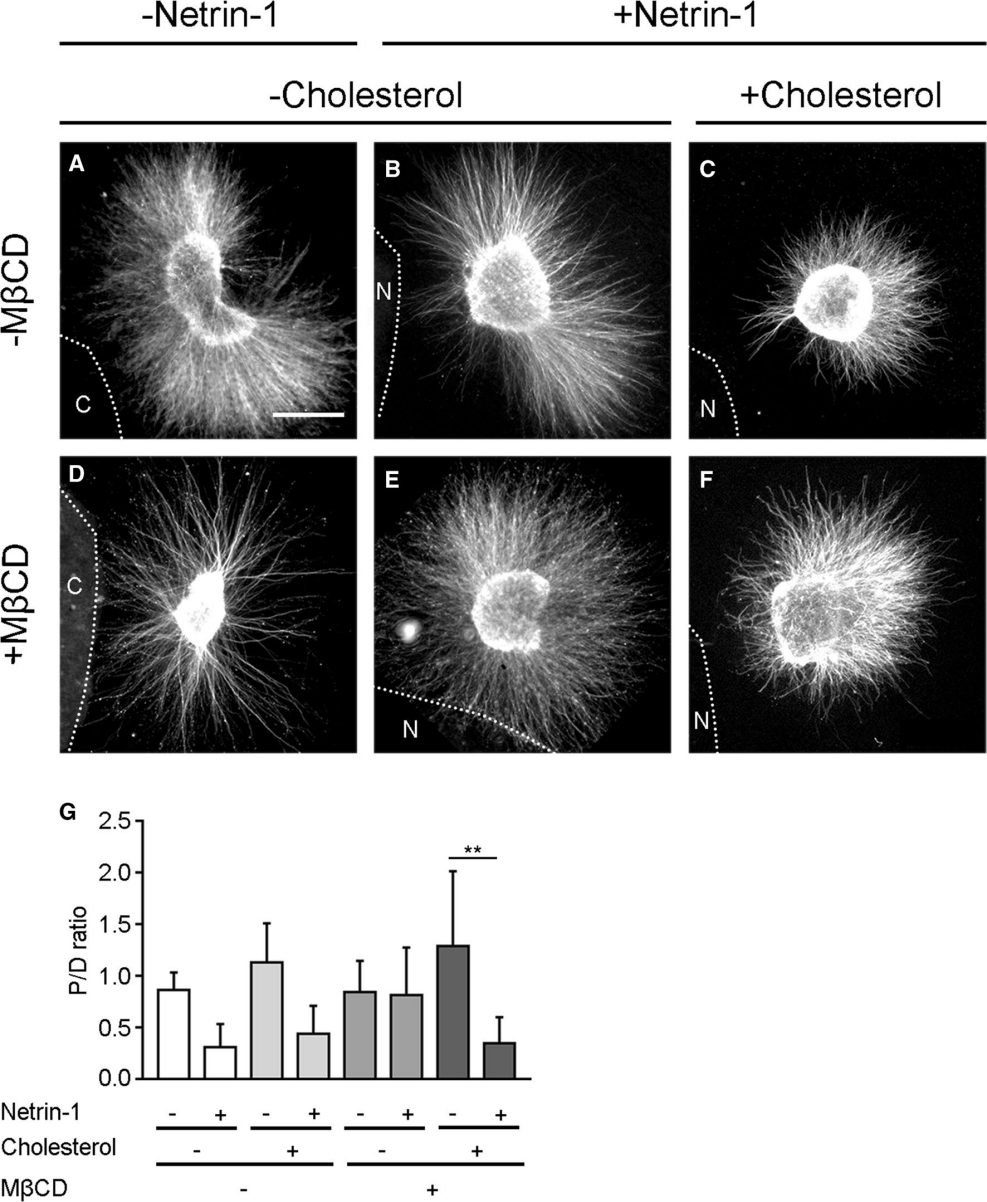
Cholesterol depletion blocks Netrin-1-induced repulsion in EGL explants. **a**-**f** Representative images of EGL explants from P4 mice, immunodetected with anti-tubulin beta-III. Explants confronted with **a**, **d** control cell aggregates or **b**, **c**, **e**, **f** Netrin-1 aggregates. **a**-**c** Explants not treated with the lipid raft disrupting drug MβCD or **d**-**f** treated with MβCD. Cholesterol levels not further altered in **a**, **b**, **d**, **e** or restored in **e**, **f**. HEK-293AD aggregates are outlined with a dashed line. Scale bar represents 100 µm. **g** Graphs show the calculated P/D ratio. Data represent mean ± SD. One-way ANOVA followed by Tukey's multiple comparison post hoc test was used. **p ≤ 0.01

We next analysed the extension of axonal guidance processes from cerebellar EGL explants grown embedded in type I collagen 3D hydrogels, and exposed to aggregates of either control HEK-293AD cells or HEK-293AD cells expressing Netrin-1 [26]. The formation of a gradient of secreted Netrin-1 along the hydrogel causes significant axonal repulsion of growing EGL processes [13]. Some EGL explants were treated with MβCD (1 mM, 30 min) after being embedded in the collagen matrix. After 2DIV, explants were fixed and labelled with tubulin-βIII to identify axonal projections (Fig. 9a-f). In the absence of MβCD, explants confronted with control cells exhibited radial outgrowth (Fig. 9a, d), whereas those confronted with Netrin-1-expressing cells showed significant repulsion (Fig. 9b, c, f). Treatment of explants with MβCD did not affect the normal radial extension of axons in control conditions (Fig. 9d) but did reduce the chemorepulsive response to Netrin-1 (Fig. 9e). These results were analysed by measuring the proximal/distal (P/D) ratio (Fig. 9g). To corroborate the specificity and reversibility of MβCD-mediated cholesterol depletion, endogenous cholesterol was restored by incubation with exogenous cholesterol immediately after MβCD treatment. Netrin-1-dependent axon chemorepulsion was restored by adding cholesterol to the medium (Fig. 9c, f). The loss of Netrin-1-induced growth cone collapse and repulsion of EGL axons, after incubation with drugs that decrease cholesterol membrane levels, indicates the importance of this lipid during Netrin-1-dependent axon guidance in EGL neurons.

EGL neurons express both DCC and UNC5 Netrin-1 receptors and we have shown that both proteins are located in raft microdomains. To evaluate the importance of DCC in EGL axonal chemorepulsion to Netrin-1, we specifically blocked DCC by incubating EGL explants with anti-DCC monoclonal antibody [27]. The presence of anti-DCC antibodies did not affect the observed chemorepulsion, suggesting that DCC receptors are not functionally relevant during Netrin-1-dependent repulsion of EGL axons (Supplementary Fig. 4a–e).

### Repulsion mediated by Netrin-1 is abolished after CYP46A1 depletion

**Fig. 10:**
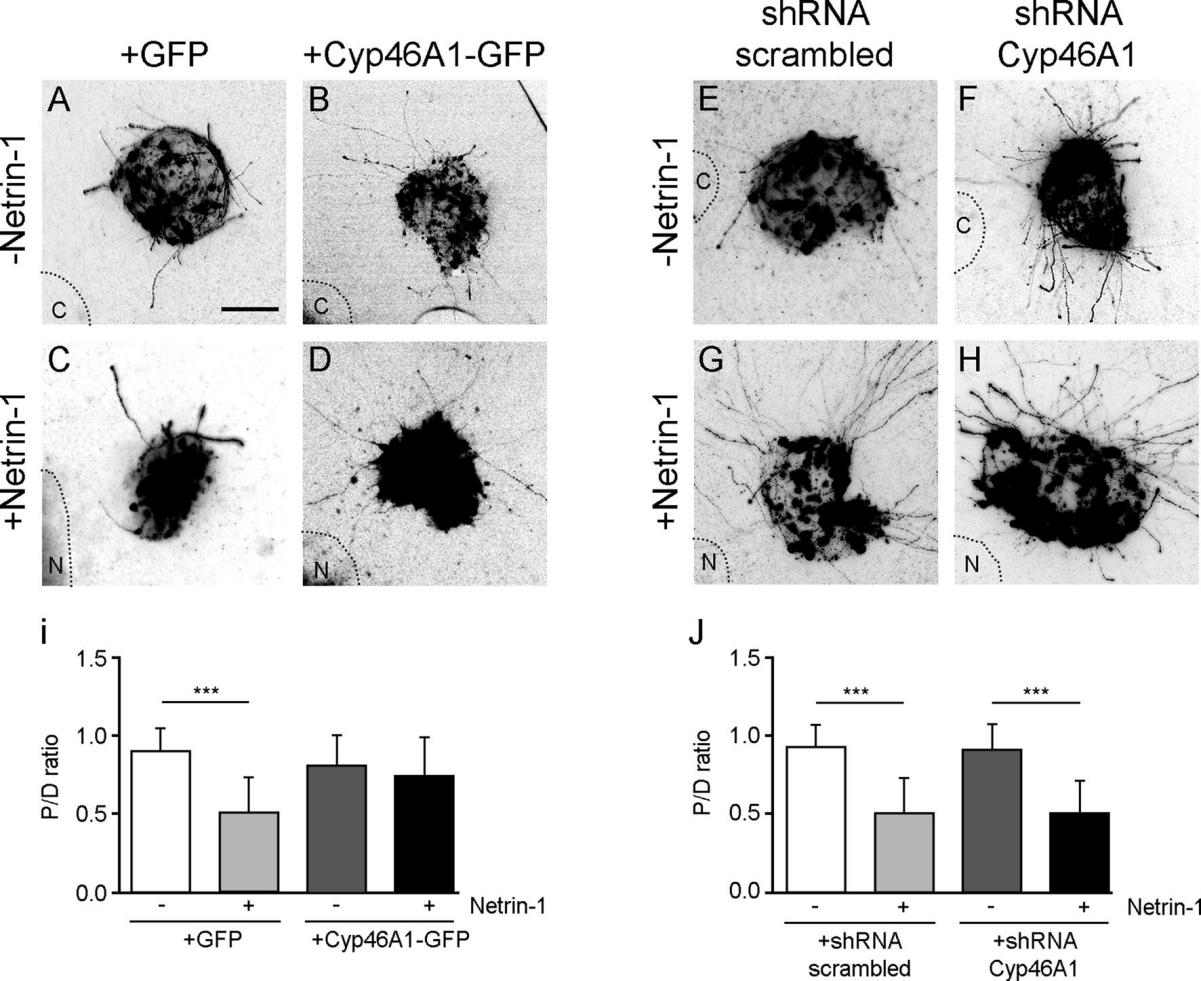
Genetically modified levels of cholesterol shift repulsion into radial axonal outgrowth in EGL explants. **a**-**h** Representative images from EGL explants from P4 mice. Explants were electroporated with a GFP control plasmid in **a** and **c**; a Cyp46A1 overexpression plasmid in **b** and **d**; a scrambled shRNA plasmid in **e** and **g;** or a shRNA plasmid against Cyp46A1 in **f** and **h**. EGL explants were confronted with HEK-293AD control cell aggregates in **a**, **b**, **e** and **f;** or were confronted with HEK-293AD-Netrin-1 expressing cell aggregates in **c**, **d**, **g** and **h**. HEK-293AD aggregates are outlined with a dashed line. Scale bar represents 100 µm. **i** Graph representing calculated P/D ratios. **j** Graph representing calculated P/D ratios. Data represent mean ± SD. One-way ANOVA followed by Tukey's multiple comparison post hoc test was used. ***p ≤ 0.001

To further characterize the importance of cholesterol-dependent raft microdomains in the repulsion against Netrin-1, we genetically modulated neuronal cholesterol levels by altering the expression of the enzyme 24S-cholesterol hydroxylase (Cyp46A1). Cyp46A1 is a cholesterol-catabolic enzyme that converts cholesterol to (24S)-24-hydroxycholesterol [28]. It is abundantly expressed in the brain where it controls cholesterol efflux by transforming the impermeant cholesterol to (24S)-24-hydroxycholesterol that can efficiently pass the blood–brain barrier to be secreted into the circulation [29]. Whereas overexpressing Cyp46A1 decreases neuronal cholesterol [30], downregulation of the expression of Cyp46A1 in hippocampal neurons increases its concentration [30, 31]. We transfected EGL explants with plasmids expressing Cyp46A1 or an empty vector. Non-transfected neurons exhibited a normal repulsive response to Netrin-1. Although the final levels of cholesterol were not measured in Cyp46A1-expressing neurons inside the explants, the analysis of neurons overexpressing Cyp46 revealed decreased repulsiveness against Netrin-1 (Fig. 10a–d, i). In contrast, using a short hairpin RNA directed against mouse Cyp46A1 mRNA we evaluated the effect of increasing cholesterol levels on Netrin-1 chemorepulsion. A reduction of Cyp46A1 in EGL explants did not affect the degree of axonal repulsion against a source of Netrin-1 (Fig. 10e–h, j). Overall, these results suggest that decreasing cholesterol levels has major consequences for axonal responsiveness to Netrin-1.

## Discussion

During the development of the nervous system, extracellular cues are recognized by transmembrane receptors that can be embedded in cholesterol-enriched membrane microdomains called lipid rafts. Netrin-1 is a guidance molecule responsible for the organization of EGL neurons during early post-natal cerebellar development. UNC5 receptors lead EGL axons away from the source of Netrin-1 [13]; however, little is known about the importance of their localization in lipid rafts during cerebellar Netrin-1-dependent growth cone chemorepulsive events. Our results suggest that the distribution of UNC5 into these cholesterol-enriched raft microdomains is crucial for the Netrin-1-dependent chemorepulsion observed in EGL neurons.

UNC5 receptors are localized into rafts in heterologous expression systems where they control the initiation of cell death [18]. However, no member-specific differences in membrane distribution have been reported. Here, we describe differences in the organization and biophysical properties of the members of the UNC5 receptor family within the plasma membrane. Our results are in line with others showing that the different UNC5 receptors share a strong sequence homology within structural hallmarks such as their transmembrane domain or their DD [32], with UNC5D being the most distant member. This could explain the increased intracellular retention observed with UNC5D. UNC5A and UNC5C concentrate into large macroscopic clusters along the cell membrane, whereas UNC5B and UNC5D show homogenous membrane distribution. Accordingly, our FRAP and sptPALM experiments suggest that UNC5A and UNC5C are the least mobile receptors. Calculation of FRAP ratios provides information on the lateral mobility and diffusion of membrane proteins. Our FRAP protocol was conducted during a short period of time (240 s), fluorescence recovery was initiated at the edges of bleached ROI rather than an homogenous recovery along the entire bleached area, and all curves were fitted to a single exponential [33]. This suggests that the observed recovery was due to lateral diffusion of unbleached fluorescent protein located adjacent to the bleached region, rather than membrane insertion of new proteins from intracellular compartments. Our single-molecule analysis further confirmed the results from the FRAP experiments, showing that UNC5C receptors are less mobile than those of UNC5B. The use of conventional microscopy techniques allowed us to identify only UNC5C-forming macroclusters with lower mobility parameters. However, implementation of super-resolution microscopy revealed that both UNC5C and UNC5B are organized into nanodomains at the sub-diffractional level and that the global distribution of diffusion coefficients of their molecules is bimodal. This bimodal behaviour reflects the existence of at least two distinguishable populations. Although both of these UNC5 receptors have a predominant immobile population, UNC5B has the larger mobile population and UNC5C has the larger immobile population. As a consequence, the lower mobility of UNC5C results from increasing the population of trapped molecules, together with a decrease in the freely mobile population. As has been reported for other membrane proteins [34], the decreased lateral mobility of UNC5 receptors within nanoclusters can be attributed to an interaction with scaffolding proteins such as caveolin or PSD-95. The freely mobile UNC5 population may show a higher tendency to escape from nanodomains. Lipid composition can impact the mobility of transmembrane proteins, with cholesterol-enriched microdomains being preferentially associated with the retention of proteins in nanodomains [35]. UNC5C forms macroclusters that colocalize with CTxB-positive regions, and shows very low mobility within these structures compared with immediate adjacent regions. CTxB is a lipid raft marker and the preferential recovery of UNC5C inside raft regions suggests the existence of an ultrastructural organization responsible for its recruitment and immobilization. Lateral immobilization of UNC5 receptors is associated with their organization into cholesterol-enriched raft microdomains because (1) the mobility of UNC5C in CTxB-positive regions is reduced and (2) the immobilization is neutralized when cholesterol-enriched raft structures are pharmacologically dissociated with MβCD or ChOx.

The DD of UNC5B is involved in targeting this receptor to lipid rafts [18]. Our FRAP results for UNC5B and UNC5C show that removal of their DD results in a significant increase in the mobility of the truncated receptors compared with their full-length forms. This suggests that the absence of DDs relocalizes UNC5 away from low-mobility cholesterol-enriched membrane microdomains. The DDs of the UNC5 family members are similar to those present in ankyrin or p53-induced death domain-containing protein (PIDD) [36], which mediate the attachment of these integral membrane proteins to the spectrin-actin-based membrane cytoskeleton. Our reported differences in the sequences of UNC5 DDs might explain their specific mobility parameters due to the control of their interaction with endogenous proteins. Removal of DDs may influence the binding of UNC5 receptors to scaffolding proteins or the actin cytoskeleton, affecting their lateral membrane mobility and their interaction with cholesterol-enriched microdomains. Although we have evaluated the importance of DDs for the mobility of UNC5 receptors using FRAP, it would be interesting in the future to assess the specific amino acid sequence responsible for controlling the nanoscale organization of these receptors.

UNC5B and UNC5C are involved in the Netrin-1-dependent axonal pathfinding of granule cells during postnatal cerebellar development [13]. Our FRAP results showed that the cell membrane mobility parameters of UNC5B and UNC5C in hippocampal neurons were not significantly different from those obtained in HEK-293AD cells, probably due to the fact that HEK-293 cells are likely to be of neuronal origin [37]. Whereas the importance of raft integrity in Netrin-1-mediated neuronal function has been identified for DCC [18] and neogenin [38], the physiological function of raft microdomains during UNC5-dependent Netrin-1-mediated axon chemorepulsion has remained unexplored. To evaluate the importance of cholesterol-enriched raft microdomains for the guidance of EGL axons by Netrin-1, we depleted endogenous cholesterol using a combination of pharmacological and genetic approaches. Upon Netrin-1 binding to UNC5 receptors, the growth cones of ELG neurons collapse and their axons are repelled. However, pre-treatment with cholesterol-depleting agents such as MβCD or ChOx prevented these outcomes. Interestingly, even though the mechanisms of action for both drugs are different, they are able to undermine membrane cholesterol, resulting in the inhibition of Netrin-1-mediated chemorepulsive effects. The specificity and reversibility of cholesterol depletion with MβCD treatment is demonstrated by the restoration of Netrin-1-mediated-repulsion upon cholesterol reload. Alternatively, downregulation of the expression of Cyp46A1 in hippocampal neurons increases the concentration of cholesterol [30, 31], whereas overexpressing Cyp46A1 decreases the levels of cholesterol in neurons [30]. Only overexpression of Cyp46A1 decreased the repulsiveness by Netrin-1 in EGL neurons, reinforcing the idea that the integrity of cholesterol-enriched raft microdomains is required to maintain an optimal effect of Netrin-1 during EGL axon navigation. These results are in line with the disruption of Netrin-1 attraction in dorsal spinal cord neurons upon lipid raft destabilization [39], suggesting that, analogous to DCC, the repulsive function of UNC5 receptors depends on the integrity of cholesterol-enriched raft microdomains.

The importance of cholesterol-enriched membrane microdomains during cerebellar development is also reflected by the phenotype observed in Zellweger syndrome, a lethal inherited disorder characterized by severe defects in peroxisome biogenesis, with a resulting lower level of plasma cholesterol. Deficiency of the peroxisome assembly gene (PEX2) in mouse brain is a model of Zellweger syndrome [40]. These animals are characterized by abnormal cerebellar histogenesis, with marked abnormalities in granule neuron population [41]. This specific phenotype is in line with our results, reinforcing the idea that intact cholesterol-enriched membrane microdomains are required for the proper chemorepulsive response of EGL axons to Netrin-1.

Previous results from our team and others have shown that raft cholesterol-enriched structures are also important for axonal regeneration following spinal cord and peripheral nerve injury [38, 42, 43]. Netrin-1 is expressed in the developing and adult spinal cord by cells from the central canal and the floor plate [44]. After spinal cord injury, high levels of Netrin-1 can still be detected for 3 months [44]. The attractive function of Netrin-1 could, therefore, be used to promote peripheral regeneration. However, the predominant expression of UNC5(A–D) in adult spinal cord neurons (corticospinal and lateral motor neurons) would activate Netrin-1 repulsive signalling to slow down the rate of axon regeneration. As our results indicate, reversing the repulsive function of UNC5 receptors using cholesterol-modifying agents represents another possible mechanism by which raft disruption could promote axonal regeneration in spinal cord injury models.

Using a combination of biochemical and imaging techniques we describe a previously unknown differential distribution of UNC5 Netrin-1 receptors, with some members being more immobilized than others into cholesterol-enriched raft microdomains. Importantly, using both pharmacological and genetical approaches, we demonstrate the physiological importance of this membrane organization, with the localization of UNC5 in lipid rafts appearing to be crucial for Netrin-1-dependent chemorepulsive events during early postnatal development of cerebellar EGL neurons. These results elucidate the importance of the organization of proteins into specific membrane microdomains and the potential implications for the development of novel therapies against pathological conditions such as spinal cord lesions.

## Materials and methods

### Antibodies, drugs and reagents

The following antibodies were used: polyclonal rabbit anti-IIIβ-tubulin (MMS-435P; Covance), polyclonal rabbit anti-UNC5B (ab104871; Abcam), polyclonal goat anti-UNC5C (ab106949; Abcam), polyclonal rabbit anti-caveolin-1 (ab2910; Abcam), monoclonal mouse anti-clathrin heavy chain (ab2731; Abcam), polyclonal rabbit anti-GFP (A11122, Invitrogen-Thermo Fisher Scientific), wheat germ agglutinin (WGA) Alexa Fluor 594-conjugated (W11262; Invitrogen-Thermo Fisher Scientific), cholera toxin B-subunit (CTxB) Alexa Fluor 488-conjugated (C22841; Invitrogen-Thermo Fisher Scientific), Alexa Fluor 488-conjugated donkey anti-goat (A11055; Invitrogen-Thermo Fisher Scientific) and Alexa Fluor 488-conjugated goat anti-rabbit (A32731; Invitrogen-Thermo Fisher Scientific). The HRP-labelled secondary antibodies were goat anti-mouse immunoglobulins/HRP (P0447; DAKO) and goat anti-rabbit immunoglobulins/HRP (P0448; DAKO).

The following drugs and reagents were used: poly-d-lysine (P7280; Sigma-Aldrich), methyl-β-cyclodextrin (C4555; Sigma-Aldrich), phalloidin-TRITC (P1951; Sigma-Aldrich), DMSO (D5879; Sigma-Aldrich), DAPI (D9542; Sigma-Aldrich), recombinant mouse Netrin-1 (1109-N1-025; R&D Systems, Inc), rat tail collagen type I (354236; BD Biosciences), cholesterol oxidase *Streptomyces sp*. (228250; Calbiochem).

### Plasmids

UNC5(A–D) full-length receptors were subcloned into a pEYFP plasmid (6004-1; Clontech), with EYFP placed at the C terminus of each receptor. With UNC5A using flanking restriction sites *HindIII* and *EcoRI* (*forward primer* 5′ CCC AAG CTT ATG GCC GTC CGG CCC GGC CTG and *reverse primer* 5′ CCG GAA TTC TCC GCA CTC GGC CTC TGA CAC), UNC5B using flanking restriction sites *HindIII* and *EcoRI* (*forward primer* 5′ CCC AAG CTT ATG AGG GCC CGG AGC GGG GTG and *reverse primer* 5′ CCG GAA TTC TCC GCA ATC GCC ATC TGT GG), UNC5C using flanking restriction sites *XhoI* and *EcoRI* (*forward primer* 5′ CCG CTC GAG ATG AGG AAA GGT CTG AGG GC and *reverse primer* 5′ CCG GAA TTC TCC ATA CTG TCC TTC TGC TG), UNC5D using flanking restriction sites *NheI* and *HindIII* (*forward primer* 5′ CTA GCT AGC ATG GGG ACA GGG GCT GCA GAC G and *reverse primer* 5′ CCC AAG CTT TAG AGT CCA TTT TGC CTG CTG) or into a pmCherry plasmid only for the UNC5C gene using the same pair of primers mentioned above. UNC5B and UNC5C were also subcloned into the pmEos2 vector (Addgene #54662) to perform sptPALM experiments. mEos2 was placed at the C terminus of both molecules, UNC5B was subcloned using *EcoRI* and *SalI* restriction sites (*forward primer* 5′ GGA ATT CCG ATG AGG GCC CGG AGC GGG GTG and *reverse primer* 5′ CGC GTC GAC GTG CAA TCG CCA TCT GTG GCC AT) and UNC5C was subcloned using *NheI* and *XhoI* restriction sites (*forward primer* 5′ GGC TAG CCA TGA GGA AAG GTC TGA GGG CG and *reverse primer* 5′ GGC TCG AGA TAC TGT CCT TCT GCT GCC AA). Inserts were purchased (Source Bioscience) and subcloned into the mentioned vectors. Primers used for site-directed mutagenesis were *forward primer* 5′ CTG TCC ATC CGC CAA GAA TTC TGC AGC AGC CTG and *reverse primer* 5′ CAG GCT GCT GCA GAA TTC TTG GCG GAT GGA CAG for UNC5B targeting the 2560 position to create a *EcoRI* site and delete the DD (UNC5B-ΔDD) and *forward primer* 5′ CCC TAT CCG GCA GGA ATT CTG CAG CAG CCT GGA TG and *reverse primer* 5′ CAT CCA GGC TGC TGC AGA ATT CCT GCC GGA TAG GG for UNC5C targeting the 2594 position to create a *EcoRI* restriction site and delete the DD (UNC5C-ΔDD). The pmCherry vector was a gift from Dr. Francesc Tebar (University of Barcelona, Spain). pCDNA3-hDCC was generously provided by Prof. Elke Stein (Yale University, United States) and subcloned into a pEYFP plasmid using *NheI* and *XhoI* as the flanking regions (*forward primer* 5′ CTA GCT AGC ATG GAG AAT AGT CTT AGA TG and *reverse primer* 5′ CCG CTC GAG TAA AAG GCT GAG CCT GTG ATG) shRNA against Cyp46A1, shRNA scrambled control and Cyp46a1-GFP were a generous gift from Prof. Carlos Dotti (Centro de Biología Molecular Severo Ochoa CSIC/UAM, Spain).

### Heterologous cell cultures

HEK-293AD cells were maintained in DMEM (11995065; GIBCO-Thermo Fisher Scientific) medium supplemented with 10% fetal bovine serum (FBS) (26140079; GIBCO-Thermo Fisher Scientific), 1% GlutaMAX (35050061; GIBCO-Thermo Fisher Scientific) and 1% penicillin/streptomycin (15140122; GIBCO-Thermo Fisher Scientific).

### Primary neuronal cultures

CD1 pregnant females and postnatal mice were used for the experiments (Charles River). Primary cultures of cerebellar EGL neurons were prepared from P4-P5 postnatal mice. Animals were sacrificed by decapitation in accordance with relevant institutional and governmental ethical guidelines and regulations. Cerebellums were isolated, mechanically disaggregated and trypsinized as previously described [43]. After centrifugation, neurons were resuspended in 2 mL of DMEM medium and the EGL was isolated by centrifugation (3000 rpm, 10 min at 4 ℃) in a percoll gradient (35% and 60% of percoll). After washing with PBS, EGL neurons were plated on poly-d-lysine pre-coated dishes in plating medium. The composition of the plating medium was DMEM, 1% penicillin/streptomycin, 1% glutamine, 4.5% d-(+)-glucose (G-8769; Sigma), 5% NHS (26050–088; GIBCO-Thermo Fisher Scientific), 10% FBS. The plating medium was maintained for 24 h and then changed to the culture medium, which had the same composition as the plating medium except for NHS and FBS, which were replaced by 2% B27 (17504001; GIBCO-Thermo Fisher Scientific) and 1% N2 (11520536; GIBCO-Thermo Fisher Scientific).

EGL explants were obtained from P4-P5 mice. The animals were sacrificed by decapitation, after which the cerebellums were isolated and chopped in 300 µm slices. Selected slices were further dissected using fine tungsten needles to extract small tissue pieces from the EGL. Explants were carefully placed inside a 3D collagen matrix prepared as previously described [26]. The collagen matrix was formed on poly-d-lysine pre-coated dishes and cultured in culture medium for 24 h.

Primary cultures of mouse hippocampi were prepared from E16-E17 embryos. Pregnant CD1 mice were sacrificed by cervical dislocation, and the foetuses were collected in a PBS-glucose 0.3% solution. Hippocampi were isolated and trypsinized for 6 min at 37 ℃. The trypsin was then neutralized with FBS and incubated with DNase for 10 min at 37 ℃. Neurons were centrifuged at 800 rpm for 5 min, resuspended and plated in pre-coated culture glasses (Nunc) with poly-d-lysine in medium containing Neurobasal medium (without l-glutamine) (21103049; GIBCO-Thermo Fisher Scientific).

### Transfection of HEK-293AD cells and neurons, and electroporation of explants

One day prior to transfection, cells were counted and plated in previously PDL-coated 35 mm coverslips, to have a 70% of confluence on the day of transfection. HEK-293AD cells were transfected with Lipofectamine 2000 (11668019; Thermo Fisher Scientific) (4 µg of DNA and 8 µL of Lipofectamine 2000) following the manufacturer’s instructions.

After 2DIV, the cultures were transfected with UNC5(B-D)-YFP, using Lipofectamine 2000. Prior to the transfection procedure, 2:5 of medium was removed and kept aside to be returned after transfection. For each 35 mm plate, 4 µg of DNA and 8 µL of Lipofectamine 2000 were used. The final mixture of DNA- Lipofectamine 2000 was carefully added to the cultures and further incubated at 37 ℃ in 5% CO_2_ for 50 min. The medium was replaced for that removed at the beginning and an equal amount of fresh Neurobasal medium (1% penicillin/streptomycin, 1% glutamax, 2% B27). Cultures were incubated until the following day.

Explants were electroporated with the Invitrogen Neon system. Briefly, dissected explants were washed three times in PBS and suspended in buffer R with 5 µg of DNA. Conditions of the electroporation were voltage: 500 V; width: 50 ms; 5 pulses. Explants were incubated for 1 h in Neurobasal medium and then mounted in a 3D collagen matrix.

### Immunocytochemistry

HEK-293AD cells and primary neurons were fixed with a solution of 4% paraformaldehyde (PFA) in PBS for 10 min at room temperature. Neuronal explants were fixed by incubation with the same solution for 30 min at room temperature. Cells were rinsed with PBS and permeabilized with a solution of 0.1% Triton-X-100 in PBS for 10 min, or 30 min for the explants. Cells were then washed with PBS and incubated in blocking solution (10% NHS in PBS) for 1 h at room temperature, or 3 h for the explants. After blocking, the cells were incubated with the respective primary antibodies diluted in blocking solution for 2 h at room temperature, or overnight at 4 ℃ for the explants. Cells and explants were washed with PBS and incubated in the secondary antibody solution (PBS 1 × with 10% NHS) for 1 h at room temperature, or 3 h for the explants. Finally, cells and explants were washed and mounted in Mowiol (81381; Sigma-Aldrich) for imaging.

Colocalization of UNC5 receptors with the WGA membrane marker and CTxB lipid raft membrane marker was performed at 4 ℃ for 30 min to avoid internalization of the marker. Briefly, colocalization was quantified by performing thresholding of the corresponding channels (UNC5, WGA or CTxB) to obtain binary masks that overlapped. Colocalization was considered as the fraction of UNC5 area that overlap with the membrane or the raft marker.

### FRAP analysis of UNC5 membrane dynamics

FRAP experiments were performed on different days but always following the same protocol. Cells expressing the YFP-tagged receptor were placed in a cell chamber Attofluor, with preheated 37 ℃ imaging medium (MEM, 30 mM HEPES, pH 7.4). Living cells were always in the incubator at 37 ℃ and 5% CO_2_. Cells were imaged using a iXon EMCCD Andor DU-897 camera with a 100x/1.4 oil objective. Cells were excited with a 488 nm diode laser at 25%. The FRAP protocol was divided into three sections: initially cells were scanned 50 times every 0.4 s at three different Z levels (Δ 0.75 μm) (prebleach). Then, a small selected region of the cytoplasmic membrane was photobleached, using a 488 nm diode laser at 80% and a ROI line (10 μm) (dwell time: 60 μs, 10 repeats) and scanned 300 times at maximum speed (one frame every 0.136 s) (postbleach). This was termed the fast acquisition phase. Thereafter, cells were scanned 90 times every 2 s (postbleach) at three different Z levels (Δ 0.75 μm). Fiji software was used to measure intensities from the recordings, while IGORpro v6.0 (WaveMetrics) software was used to process the measurements. Three different measurements for each time point were taken with Fiji: whole-cell integrated density, background integrated density and fluorescence recovery integrated density. The data was then analysed with IGORpro v6.0 (WaveMetrics). First, recovery intensities were normalized to 1, taking the prebleach intensities as absolute. Whole-cell and recovery intensities were corrected by subtracting background intensities. Finally, whole-cell intensities were used to correct the recovery intensities, needed to correct for photobleaching due to the acquisition protocol. This is the formula used to correct and normalize the FRAP curve to 1:

$$I_{FRAP norm} \left( t \right) = \frac{{I_{whole - prebleach} }}{{I_{whole} \left( t \right) - I_{background} \left( t \right)}} \times \frac{{I_{FRAP} \left( t \right) - I_{background} \left( t \right)}}{{I_{FRAP - prebleach} }}$$

Once the normalized points were calculated at each time point, averages and SD values for each condition were plotted in a graph and the FRAP curve was then fitted to a single exponential. *y*_0_ is the *y* value at the photobleaching point, τ is the time constant and *A* is the amplitude of each component. Finally, the mobile fraction (Mf) was calculated as:

$$I\left( t \right) = y_{0} + Ae^{ - \tau t}$$

$$M_{f } = \frac{ - A}{{1 - \left( {y_{0} + A} \right)}}$$

The halftime (t_1/2_) was the time when the recovery of the fluorescence was half of *A*. t_1/2_ was calculated with the substitution of f(t) by *A*/2, which finally allowed us to calculate t_1/2_ according to the following formula:

$$t_{1/2} = \frac{\ln 0.5}{{ - t}}$$

### sptPALM experiments

sptPALM experiments were performed on an Elyra PS1 STORM/SIM microscope (Carl Zeiss Microscopy GmbH) equipped with a 100 × objective (α Plan-Apochromat 100 × 1.46 NA oil-immersion), a focus lock system and an EMCCD camera Andor iXon Ultra 897 (Andor Technologies). A LF488/561-A-000 beam splitter and a FF01–523/610–25 emission filter (Semrock) were used to record SEpH and mEos2 fluorescence. Cells were imaged in total internal reflection microscopy (TIRFM) or highly inclined illumination mode in an enclosed chamber at 36.0 ± 1.5 ℃.

All sptPALM measurements were performed as previously described [45]. A 560 nm laser was adjusted to 9–14 W/cm^2^. mEos2 molecules were continuously converted with a 405 nm laser, the power of which was gradually increased to maintain the density of photoconverted molecules at a low level. This ensured that only single mEos2 molecules were converted, well separated and imaged while keeping their number constant. The density of fluorescent particles was kept below 0.2 molecules/μm^2^. Images were acquired every 0.032 s.

Single-molecule fluorescent spots were localized in each frame and their position was followed over time using the TrackMate tracking algorithm [46]. A custom-written MatLab (Math Works) routine was used to visualize individual trajectories and their corresponding mean-squared displacement (MSD) plots and to generate an average MSD vs. lag time plot. To improve the relative statistical error of individual MSD curves, only trajectories with 15 points or more were included in the analysis. It has previously been shown that when the diffusion coefficient is calculated from a linear fit to the first four points of the MSD vs. time plot, the relative error of the fit increases as the number of points decreases. As a consequence, the size of the diffusion coefficient distribution increases and the range of measured diffusion coefficients can still differ by a factor of two. In such cases, the best estimate for the diffusion coefficient corresponds to the mean value [47, 48]. We, therefore, calculated the diffusion coefficients from the mean MSD vs. time plot of all trajectories recorded per cell.

The diffusion coefficient was calculated by fitting the first four points of the average MSD curve to the equation:

$$MSD\left( t \right) = 4Dt + b$$

where *t* is time and *D* is the diffusion coefficient. The average localization precision was of 40–60 nm.

To further classify UNC5 receptor motion as immobile, confined or free we performed transient mobility analysis using the Divide-and-Conquer Moment Scaling Spectrum (DC-MSS) algorithm [49]. Briefly, the DC-MSS algorithm analyses tracks in three steps: (1) it uses a local movement descriptor throughout a track to divide it into segments (tracklets) of putatively different motion classes. For this the algorithm uses a rolling window of 11 frames to reveal the switches between different motion types; (2) it classifies these segments via MSS analysis that accounts for molecule displacements; and (3) it uses the MSS analysis results to refine the track segmentation and classification. This strategy uncouples the initial identification of motion switches from motion classification. To increase the accuracy of classification, only the tracks consisting of 20 or more points were taken into account in this analysis.

### Growth cone collapse experiments

EGL primary cultures were cultured for 3DIV and then treated with MβCD (0.5 mM) for 10 min, CO (2 U) for 2 h, or the respective controls (PBS for MβCD and DMSO for CO). The neurons then were incubated with Netrin-1 (300 ng/mL) or the control (BSA 0.1%) for 45 min at 37 ℃ in culture medium (DMEM). After this incubation, neurons were fixed with 4% PFA and permeabilized with PBS-Triton-X-100 (0.1%). Finally, actin filaments were stained by incubation with phalloidin-TRITC (1 µM) for 30 min. Cells were mounted on Mowiol and used for imaging. Actin staining was used to identify growth cones, which were outlined based on differential staining for actin in this compartment with respect to the adjacent axon. An intensity threshold mask was created using Fiji [50] and the growth cone perimeter was selected using the wand tool. Collapsed growth cones were manually identified based on their morphology. In contrast to normally extended growth cones, collapsed growth cones lose their morphology, acquiring a shrivelled, round-tipped pencil-like shape devoid of lamellae or filopodia.

### Repulsion experiments

During the plating procedure, explants were embedded into the 3D collagen matrix and confronted at a distance of (200–600 µm) with aggregates of Netrin-1 stably expressing HEK-293AD cells [51]. Netrin-1 expression in stable cells was regularly tested by western blot (data not shown). In the experiments where membrane cholesterol was removed, the explants were plated and 1 h later treated with MβCD (1%) for 30 min. After the treatment, explants were washed three times with culture medium, then placed back in the normal culture medium. To replace removed cholesterol, MβCD-treated explants were grown in culture medium supplemented with cholesterol (5 mM). Explants were cultured for 48 h (2DIV). They were then fixed and immunocytochemistry against β-III tubulin was performed. Repulsion was analysed by measuring the proximal/distal (P/D) ratio. The P/D ratio is the fraction of axons growing in the proximal quadrant of the explant (closer to the aggregate of HEK-293AD cells) versus axons in the distal quadrant (far from the aggregate of HEK-293AD cells). A ratio close to 1 indicates a radial pattern of growth, below 1 indicates chemorepulsion and above 1 indicates chemoattraction. The axons located 50 μm away from the explant and extended over a line of 150 μm in the P or D quadrants.

### Sucrose gradient lipid raft isolation

HEK-293AD cells were used for raft isolation. Cells were transfected with either UNC5B or UNC5C constructs. 48 h after transfection, cells were washed twice in cold PBS and then homogenized in MES (2-morpholino ethanesulfonic acid)-buffered saline (34 mM MES, pH6.5 and 0.15 mM NaCl) plus 1% Triton X-100 supplemented with Complete Protease Inhibitor Cocktail (11697498001; Roche). Sucrose was then added to achieve a final concentration of 40%. A 5–30% linear sucrose gradient was layered on top and further centrifuged (39,000 rpm) for 20-22 h at 4 ℃ in a Beckman Sw41Ti rotor. A total of 12 fractions of 1 mL each were collected from the top and analysed by western blot.

Sucrose fractions were boiled in Laemmli SDS loading buffer and separated on 10% SDS-PAGE. They were transferred to nitrocellulose membranes (1620112; Bio-Rad) and blocked in 5% dry milk-supplemented 0.1% Tween 20 PBS prior to immunoreaction. Filters were immunoblotted with antibodies against GFP (1:2500), caveolin (1:5000) and clathrin (1:5000). Caveolin was used as a marker of detergent-resistant membranes (DRM) and clathrin as a marker of detergent-soluble membranes (DSM).

### Multiple sequence alignment

The following amino acid sequences from all UNC5 members were compared: NP_694771 (UNC5A). NP_084046 (UNC5B), NP_001280490 (UNC5C), and NP_694775 (UNC5D). Multiple sequence alignment was performed using the Clustal Omega program [52]. The phylogenetic trees comparing all sequences of UNC5 members and their respective transmembrane domains were obtained using distance correction with no gap exclusion. The neighbour-joining (NJ) method was used for clustering.

### Statistics

Results were analysed statistically using GraphPad Prism software (GraphPad Software, Inc). The D’Agostino and Pearson test was used to test for normality. The *χ*^2^ test was used to evaluate the distribution of the results into different categories. The unpaired two-tailed Student’s *t* test was used for comparison of two groups. For datasets comparing more than two groups, ANOVA followed by Tukey's multiple comparison test, the Holm-Šídák test or the Mantel-Cox test was used with corrections for multiple comparisons. Statistical comparisons were performed on a per-cell basis. The cells or neurons were randomly selected within the dishes and blindly analysed, and were collected from at least three independent experiments. Values are represented as the mean ± SD or mean ± SEM. The tests used are indicated in the respective figure legends. A *p*-value below 0.05 was accepted as significant.

## Electronic supplementary material

Below is the link to the electronic supplementary material.

Supplementary Fig.1 Structural domains are conserved across all UNC5 family members. a Alignment of mouse UNC5A, UNC5B, UNC5C and UNC5D amino acid sequences. Corresponding amino acid residues are shaded, ranging from white (different amino acid residues) to dark grey (identical amino acid residues) The signal peptide is shaded in pink at the beginning of each sequence. Structural domains are boxed: Ig domains (yellow), TSPI repeats (red), transmembrane domain (blue) and DD (green). Multiple sequence alignment was performed using Clustal Omega program (EMBL-EBI). b Phylogenetic trees comparing full sequences of UNC5 members (UNC5) and their respective DDs (DD). Accession numbers: NP_694771, UNC5A; NP_084046, UNC5B; NP_001280490, UNC5C; NP_694775, UNC5D (TIF 31991 kb)

Supplementary Fig. 2 FRAP lateral mobility of DCC-YFP in HEK-293AD and hippocampal neurons. a, b Average recovery after photobleaching in a transfected HEK-293AD cells or b hippocampal neurons expressing DCC-YFP. c Comparison of the Mf of DCC-YFP expressed in HEK-293AD cells (n = 20) or in hippocampal neurons (n = 12). d Comparison of the Mf of DCC-YFP expressed in HEK-293AD cells (n = 20) or in hippocampal neurons (n = 12). Data represent mean ± SD. Unpaired two-tailed Student’s t test was used. ***p ≤ 0.001 (TIF 1592 kb)

Supplementary Fig. 3 Biochemical fractionation and lipid raft isolation of HEK-293AD cells expressing UNC5(A-D) receptors. a-d HEK-293AD cells transfected with UNC5B-YFP or UNC5C-YFP and incubated with control medium or MβCD-containing medium. Lipid rafts were isolated by biochemical fractionation. Immunoblots were performed against GFP (to detect YFP-tagged UNC5 receptors), caveolin or clathrin. UNC5B and UNC5C were detected in fractions 3-6, colocalizing with the raft marker caveolin. Treatment with MβCD reduced the content of lipid raft-resident proteins, including UNC5B and UNC5C. DRM, detergent-resistant membranes; DSM; detergent-soluble membranes (TIF 3831 kb)

Supplementary Fig. 4 UNC5-mediated repulsion in EGL explants is independent of DCC association. a-d Representative images of EGL explants from P4 mice, immunodetected with anti-tubulin beta-III. Explants were incubated in a, b the absence or c, d the presence of anti-DCC antibody. Explants were confronted with either a, c control HEK-293AD cell aggregates or b, d Netrin-1-expressing HEK-293AD cell aggregates. HEK-293AD aggregates are outlined with a dashed line. Scale bar represents 100 µm. e P/D ratios were calculated and plotted in a bar graph. Data represent mean ± SD. An unpaired two-tailed Student’s t test was used. **p ≤ 0.01, ***p ≤ 0.001 (TIF 2816 kb)

## Data Availability

The datasets generated during and/or analysed during the current study are available from the corresponding author on reasonable request.
