# Supplementary figures and images for: Growth cone repulsion to Netrin-1 depends on lipid raft microdomains enriched in UNC5 receptors
Source: Cell Mol Life Sci. 2020 Oct 23;78(6):2797–820. doi: 10.1007/s00018-020-03663-z (PMC8004515; doi:10.1007/s00018-020-03663-z)

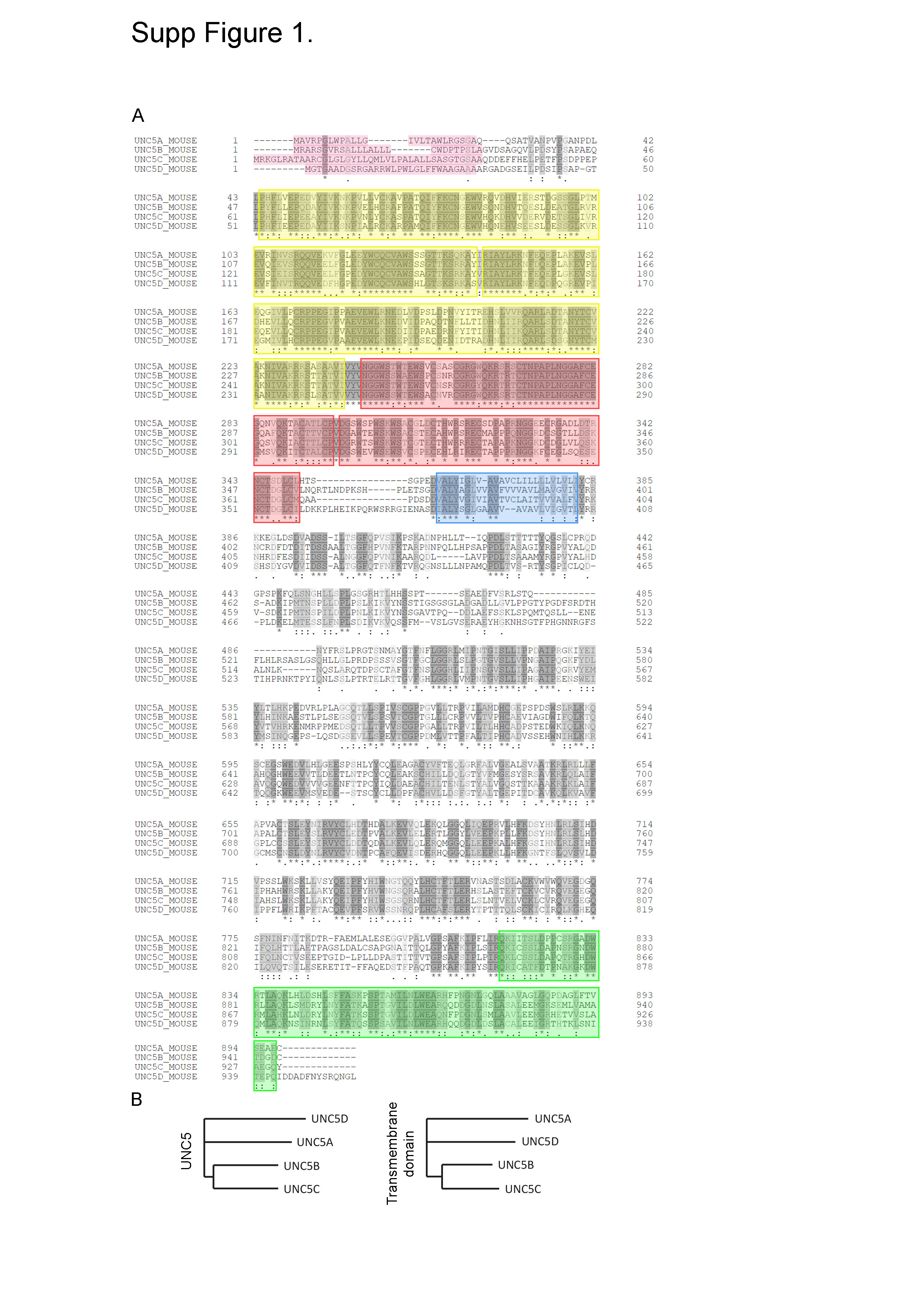

Supplement: Supplementary file 1 — Supplementary Fig.1 Structural domains are conserved across all UNC5 family members. a Alignment of mouse UNC5A, UNC5B, UNC5C and UNC5D amino acid sequences. Corresponding amino acid residues are shaded, ranging from white (different amino acid residues) to dark grey (identical amino acid residues) The signal peptide is shaded in pink at the beginning of each sequence. Structural domains are boxed: Ig domains (yellow), TSPI repeats (red), transmembrane domain (blue) and DD (green). Multiple sequence alignment was performed using Clustal Omega program (EMBL-EBI). b Phylogenetic trees comparing full sequences of UNC5 members (UNC5) and their respective DDs (DD). Accession numbers: NP_694771, UNC5A; NP_084046, UNC5B; NP_001280490, UNC5C; NP_694775, UNC5D (TIF 31991 kb) [file 18_2020_3663_MOESM1_ESM.tif]

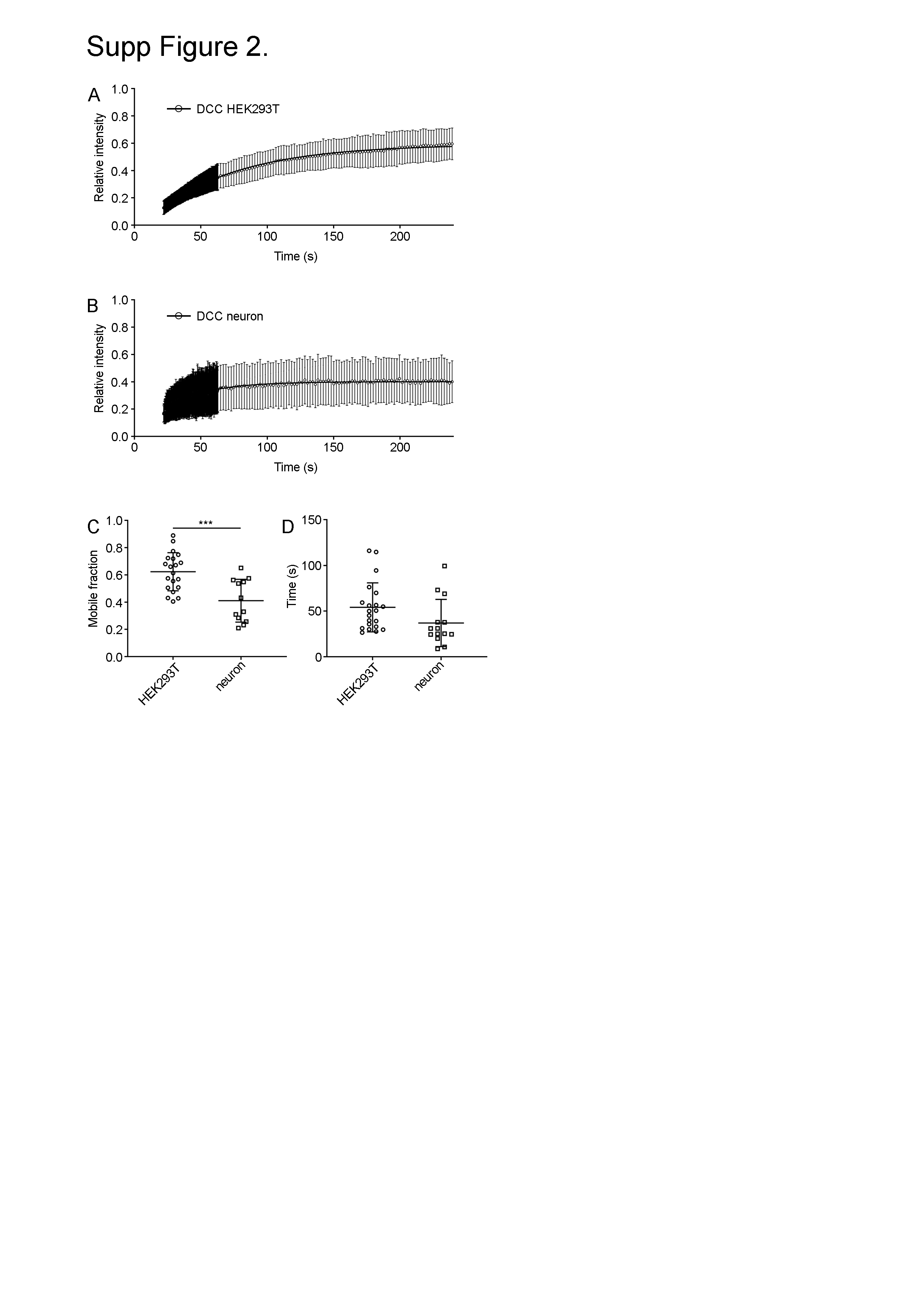

Supplement: Supplementary file 2 — Supplementary Fig. 2 FRAP lateral mobility of DCC-YFP in HEK-293AD and hippocampal neurons. a, b Average recovery after photobleaching in a transfected HEK-293AD cells or b hippocampal neurons expressing DCC-YFP. c Comparison of the Mf of DCC-YFP expressed in HEK-293AD cells (n = 20) or in hippocampal neurons (n = 12). d Comparison of the Mf of DCC-YFP expressed in HEK-293AD cells (n = 20) or in hippocampal neurons (n = 12). Data represent mean ± SD. Unpaired two-tailed Student’s t test was used. ***p ≤ 0.001 (TIF 1592 kb) [file 18_2020_3663_MOESM2_ESM.tif]

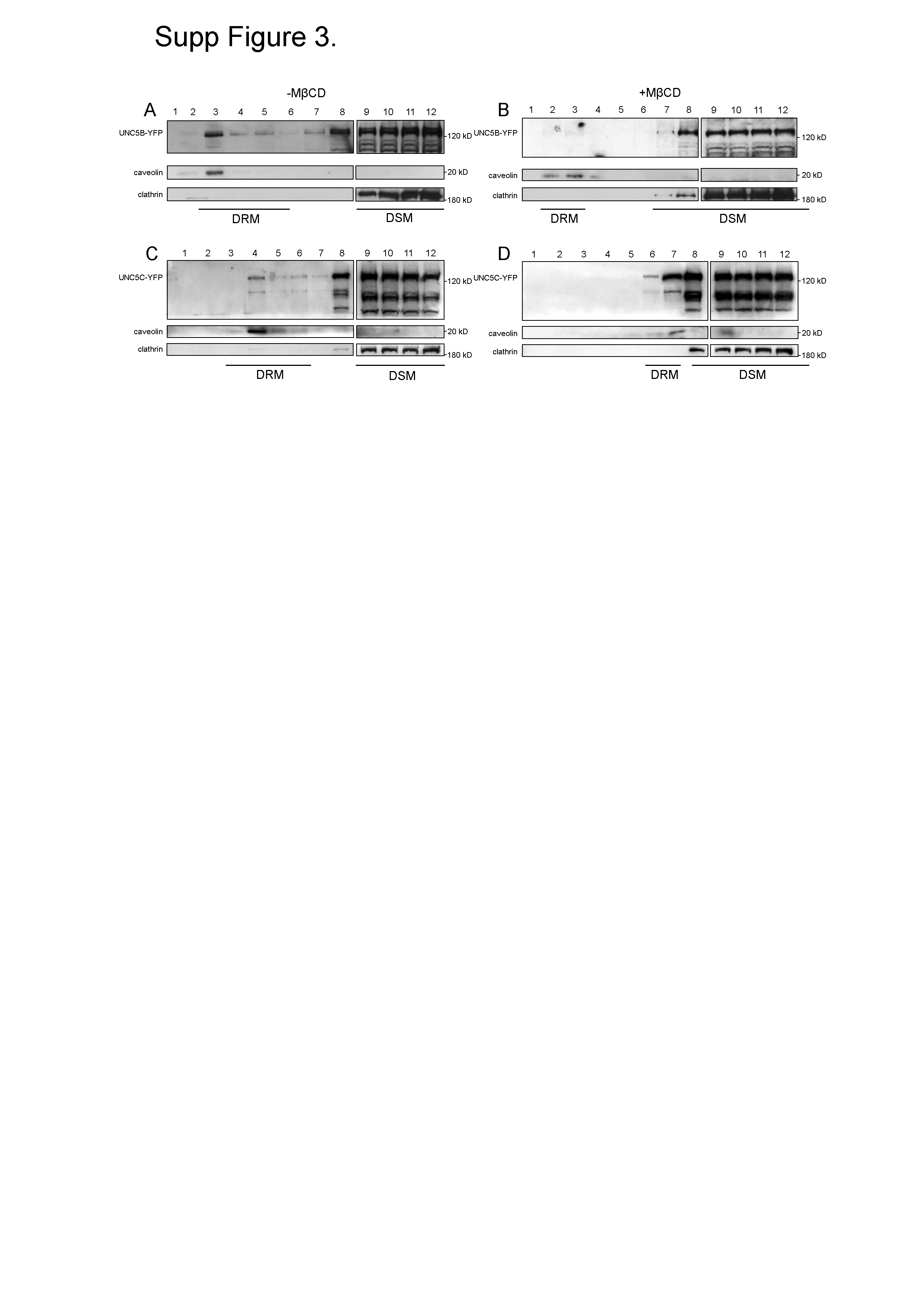

Supplement: Supplementary file 3 — Supplementary Fig. 3 Biochemical fractionation and lipid raft isolation of HEK-293AD cells expressing UNC5(A-D) receptors. a-d HEK-293AD cells transfected with UNC5B-YFP or UNC5C-YFP and incubated with control medium or MβCD-containing medium. Lipid rafts were isolated by biochemical fractionation. Immunoblots were performed against GFP (to detect YFP-tagged UNC5 receptors), caveolin or clathrin. UNC5B and UNC5C were detected in fractions 3-6, colocalizing with the raft marker caveolin. Treatment with MβCD reduced the content of lipid raft-resident proteins, including UNC5B and UNC5C. DRM, detergent-resistant membranes; DSM; detergent-soluble membranes (TIF 3831 kb) [file 18_2020_3663_MOESM3_ESM.tif]

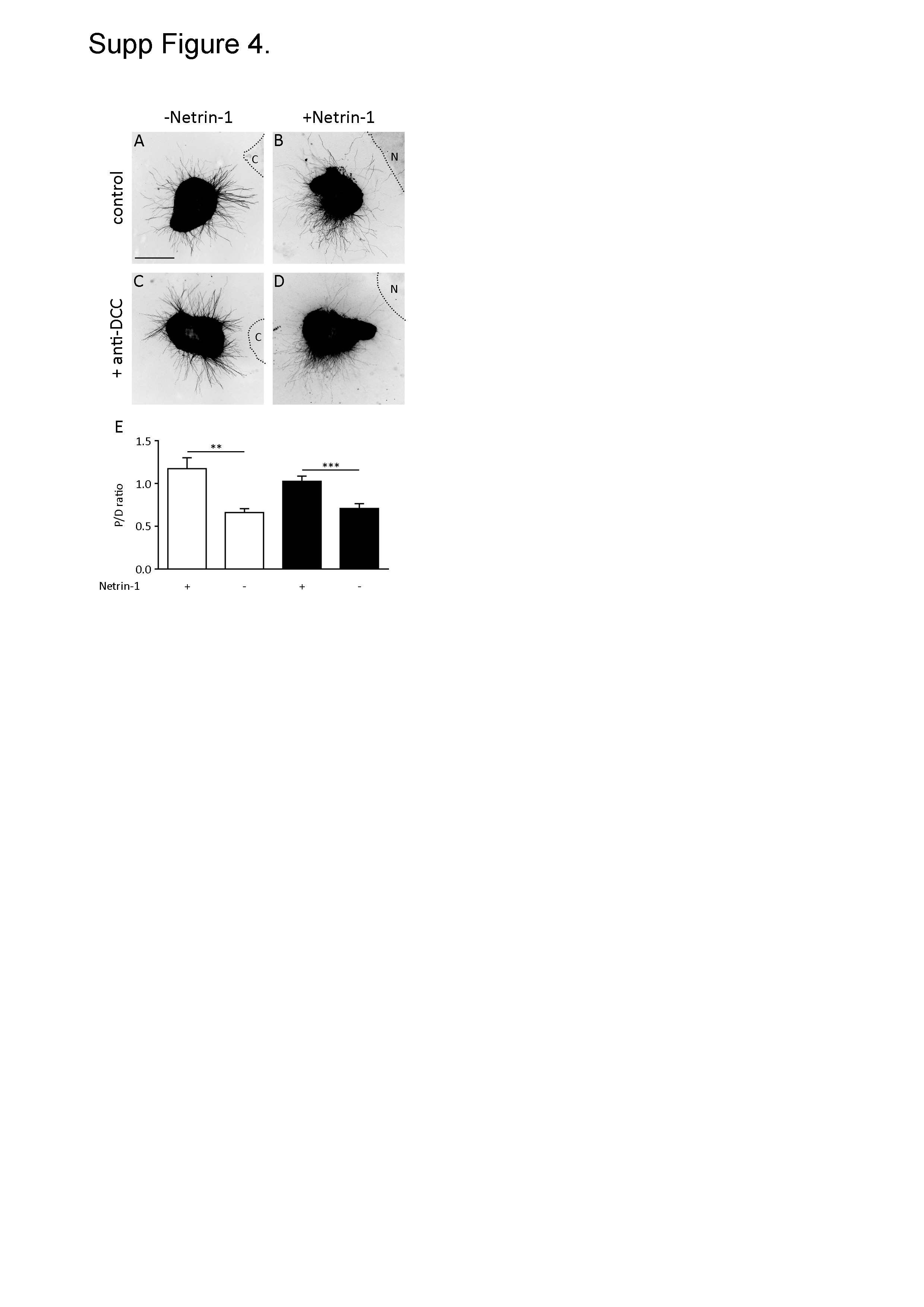

Supplement: Supplementary file 4 — Supplementary Fig. 4 UNC5-mediated repulsion in EGL explants is independent of DCC association. a-d Representative images of EGL explants from P4 mice, immunodetected with anti-tubulin beta-III. Explants were incubated in a, b the absence or c, d the presence of anti-DCC antibody. Explants were confronted with either a, c control HEK-293AD cell aggregates or b, d Netrin-1-expressing HEK-293AD cell aggregates. HEK-293AD aggregates are outlined with a dashed line. Scale bar represents 100 µm. e P/D ratios were calculated and plotted in a bar graph. Data represent mean ± SD. An unpaired two-tailed Student’s t test was used. **p ≤ 0.01, ***p ≤ 0.001 (TIF 2816 kb) [file 18_2020_3663_MOESM4_ESM.tif]
